# The role of oxygen-vacancy in bifunctional indium oxyhydroxide catalysts for electrochemical coupling of biomass valorization with CO_2_ conversion

**DOI:** 10.1038/s41467-023-37679-3

**Published:** 2023-04-11

**Authors:** Fenghui Ye, Shishi Zhang, Qingqing Cheng, Yongde Long, Dong Liu, Rajib Paul, Yunming Fang, Yaqiong Su, Liangti Qu, Liming Dai, Chuangang Hu

**Affiliations:** 1 State Key Laboratory of Organic-Inorganic Composites, College of Chemical Engineering, Beijing University of Chemical Technology, Beijing, 100029 China; 2 School of Chemistry, Xi’an Key Laboratory of Sustainable Energy Materials Chemistry, State Key Laboratory of Electrical Insulation and Power Equipment, Xi’an Jiaotong University, Xi’an, 710049 China; 3 Shanghai Advanced Research Institute, Chinese Academy of Sciences, Shanghai, 201210 China; 4 Advanced Materials and Liquid Crystal Institute, Kent State University, Kent, OH 44242 USA; 5 Department of Chemistry, Tsinghua University, Beijing, 100084 China; 6 ARC Centre of Excellence for Carbon Science and Innovation, University of New South Wales, Sydney, NSW 2052 Australia

**Keywords:** Sustainability, Electrocatalysis, Materials for energy and catalysis

## Abstract

Electrochemical coupling of biomass valorization with carbon dioxide (CO_2_) conversion provides a promising approach to generate value-added chemicals on both sides of the electrolyzer. Herein, oxygen-vacancy-rich indium oxyhydroxide (InOOH-O_V_) is developed as a bifunctional catalyst for CO_2_ reduction to formate and 5-hydroxymethylfurfural electrooxidation to 2,5-furandicarboxylic acid with faradaic efficiencies for both over 90.0% at optimized potentials. Atomic-scale electron microscopy images and density functional theory calculations reveal that the introduction of oxygen vacancy sites causes lattice distortion and charge redistribution. Operando Raman spectra indicate oxygen vacancies could protect the InOOH-O_V_ from being further reduced during CO_2_ conversion and increase the adsorption competitiveness for 5-hydroxymethylfurfural over hydroxide ions in alkaline electrolytes, making InOOH-O_V_ a main-group p-block metal oxide electrocatalyst with bifunctional activities. Based on the catalytic performance of InOOH-O_V_, a pH-asymmetric integrated cell is fabricated by combining the CO_2_ reduction and 5-hydroxymethylfurfural oxidation together in a single electrochemical cell to produce 2,5-furandicarboxylic acid and formate with high yields (both around 90.0%), providing a promising approach to generate valuable commodity chemicals simultaneously on both electrodes.

## Abstract (web summary)

Electrochemical coupling of biomass valorization with CO2 conversion provides a promising approach to generate value-added chemicals on both sides. However, bifunctional catalysts are lacking. Here the authors report oxygen-vacancy-rich indium oxyhydroxide as a bifunctional catalyst for CO2 reduction to formate and 5-hydroxymethylfurfural electrooxidation to 2,5-furandicarboxylic acid with faradaic efficiencies for both > 90.0%.

## Introduction

CO_2_ electrochemical reduction reaction (CO_2_RR) has emerged as one of the front hotspots in electrochemistry research for both the mitigation of global warming and the production of valuable chemicals^1–3^. A typical CO_2_RR testing electrode is generally paired with oxygen evolution reaction (OER) as the counter electrode with a high energy consumption due to the sluggish reaction kinetics for OER^4,5^. In addition, the O_2_ product limits the economic benefit of the electrolysis system from the view of its current value (~0.03 $/kg)^6–8^. To tackle these issues, one promising approach could be to replace the OER with the oxidizing valorization process of biomass-derived small molecules at a lower thermodynamic potential^7^, which has already been proven effective in reducing the electrolysis cell voltage for hydrogen evolution reaction (HER)^5,9,10^. By constructing an integrated electrolysis cell with coupled CO_2_RR and oxidation of biomass-derived small molecules, one could obtain not only improved overall energy efficiency but also high value-added products at both electrodes.

**Fig. 1:**
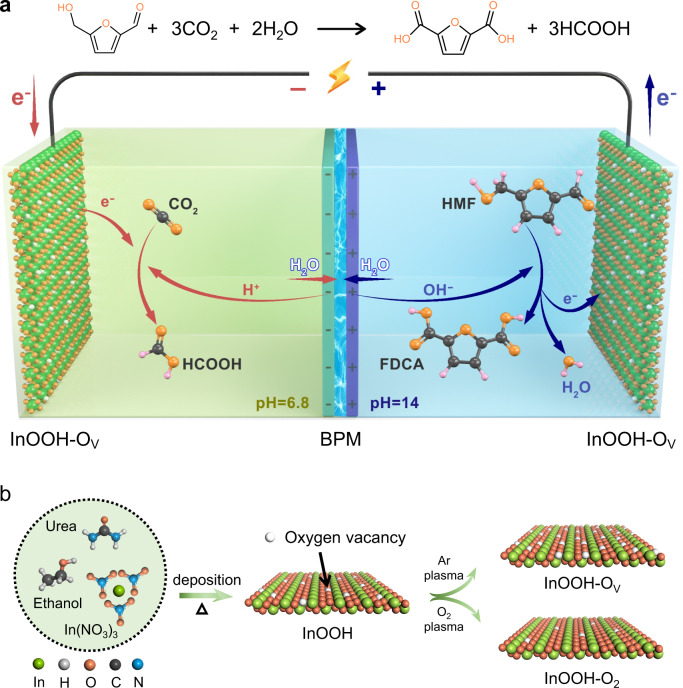
Schematic illustration. **a** Integrated electrolysis cell coupling CO_2_RR with HMFOR. **b** Synthetic processes of InOOH, InOOH-O_V_, and InOOH-O_2_.

Among the possible reduction products of CO_2_RR on the cathode, formic acid (HCOOH) is of great significance as it can serve as vital chemical intermediate in many industrial processes, a potential liquid compound for hydrogen storage, and even a fuel to be directly used in formic acid fuel cells^11–13^. For the anodic reaction, a promising candidate is the oxidation of 5-hydroxymethylfurfural (HMF), a lignocellulosic biomass-derived small molecule^14^. Owning to the presence of active hydroxyl and aldehyde groups, HMF can be transformed into various high-value chemical precursors useful for chemical industries^14,15^. Specifically, 2,5-furandicarboxylic acid (FDCA), resulting from HMF through oxidation of its two oxygen-containing groups into carboxyl, is one of the top 12 sugar-derived platform chemicals claimed by the U.S. Department of Energy^16,17^. Therefore, the electrochemical coupling of cathodic CO_2_RR with anodic HMF oxidation reaction (HMFOR) should hold great promise for synchronous production of value-added chemicals (e.g., HCOOH and FDCA) within one electrolysis cell (Fig. 1a). The half-cell and overall reactions involving in Fig. 1a are shown below.

1 $${{{{{\rm{Anode}}}}}}\,{{{{{\rm{reaction}}}}}}:{{{{{\rm{HMF}}}}}}+6{{{{{{\rm{OH}}}}}}}^{-}\to {{{{{{\rm{FDCA}}}}}}+{{{{{\rm{4H}}}}}}}_{2}{{{{{\rm{O}}}}}}+6{e}^{-}$$

2 $${{{{{\rm{Cathode}}}}}}\,{{{{{\rm{reaction}}}}}}:3{{{{{{\rm{CO}}}}}}}_{2}{+{{{{{\rm{6e}}}}}}}^{-}{+{{{{{\rm{6H}}}}}}}^{+}\to {{{{{\rm{3HCOOH}}}}}}$$

3 $${{{{{\rm{Overall}}}}}}\,{{{{{\rm{reaction}}}}}}:3{{{{{{\rm{CO}}}}}}}_{2}{+{{{{{\rm{HMF}}}}}}+{{{{{\rm{2H}}}}}}}_{2}{{{{{\rm{O}}}}}}\to {{{{{\rm{3HCOOH}}}}}}+{{{{{\rm{FDCA}}}}}}$$

To endow the integrated system with high production efficiency, two issues need to be addressed: i) An effective asymmetric electrolysis cell should be developed as a neutral electrolyte is favorable for CO_2_RR^18^ while a strong basic environment can remarkably accelerate the production of FDCA^14,19^; and ii) The activity and selectivity of the catalysts need to be upgraded to improve the production efficiencies for both HCOOH and FDCA by suppressing the corresponding competitive HER and OER reactions at cathode and anode, respectively. Thus, it is highly desirable, but still challenging, to develop a class of bifunctional catalysts for efficient CO_2_RR and HMFOR in an electrolysis cell with asymmetric pH values. If realized, the CO_2_RR and HMFOR bifunctional catalysts can simplify the electrolysis cell construction and avoid the synthesis of different catalysts, and hence energy/cost-saving for practical applications (vide infra).

Indium oxides have been demonstrated as effective electrocatalysts for CO_2_RR to generate formate with a high selectivity^20,21^, superior to most of the transition metal oxides^22,23^. In most of cases, the first-row transition metal oxides are employed as the catalysts for HMFOR^14^. However, the partially occupied d-orbitals of transition metals strongly interact with reactive functional groups of oxygen-containing molecules and intermediates, which could cause the difficulty for subsequent desorption and limit the performance of transition metal oxides for electrochemical oxidation reactions (EOR)^24^. On the other hand, main-group p-block metal oxides with the fully occupied d-orbitals and the p-bands serving as the host orbitals could facilitate the desorption of oxygenated intermediates to enhance EOR^24,25^. Different from a narrow d-band of transition metals, delocalized p-band in main-group p-block metals as the host-orbital may broaden the adsorbate state density, causing weak chemisorption and insufficient activation for reactant molecules^26,27^, which brings challenges to catalyze EORs. Till now, seldom main-group p-block metal oxides have been reported for EORs, but they indeed hold a great potential.

Herein, a plasma-aided technique was utilized to introduce the oxygen vacancies (O_V_) into indium oxyhydroxide (InOOH) nanosheets under Ar atmosphere^19,28^ by removing some of the surface lattice oxygen atoms (Fig. 1b), thus engineering the local electron environment of the adjacent indium atoms. The atomic-scale electron microscopy images along with density function theory (DFT) calculations and in-situ Raman spectroscopic analysis demonstrated that the formation of O_V_ sites cause lattice distortion and charge redistribution on the surface of InOOH nanosheets, hence enhancing the adsorption and activation of CO_2_ and HMF molecules (vide infra), which is vital to proceeding the subsequent electrochemical catalytic reactions. The resultant InOOH-O_V_ exhibited facilitated kinetics of CO_2_RR into HCOOH, leading to a high Faraday efficiency (FE) of 92.6% for formate at −0.85 V vs. reversible hydrogen electrode (RHE, the same hereinafter unless otherwise specified) along with a maximum formate partial current density (j_formate_) of 56.2 mA cm^−2^ at −1.00 V. Meanwhile, InOOH-O_V_, as main group p-block metal oxide, showed greatly promoted activity for HMFOR, achieving a high FDCA yield of 91.6% at 1.48 V. These findings prompted us to use the resultant InOOH-O_V_ as the bifunctional catalyst for integration of CO_2_RR and HMFOR in a single electrolysis cell with asymmetric pH values (Fig. 1a). A bipolar membrane (BPM) was employed to address the mismatched pH values between the anodic electrolyte (1 M KOH) for HMFOR and cathodic electrolyte (0.1 M KHCO_3_) for CO_2_RR. An integrated cell based on the InOOH-O_V_ bifunctional catalyst exhibited an anodic yield of 87.5% for FDCA and a cathodic FE around 90.0% at a cell voltage of 2.27 V for the formate, demonstrating the great promise for a combination of biomass valorization and CO_2_ conversion reactions to simultaneously produce value-added chemicals.

## Results and discussion

**Fig. 2:**
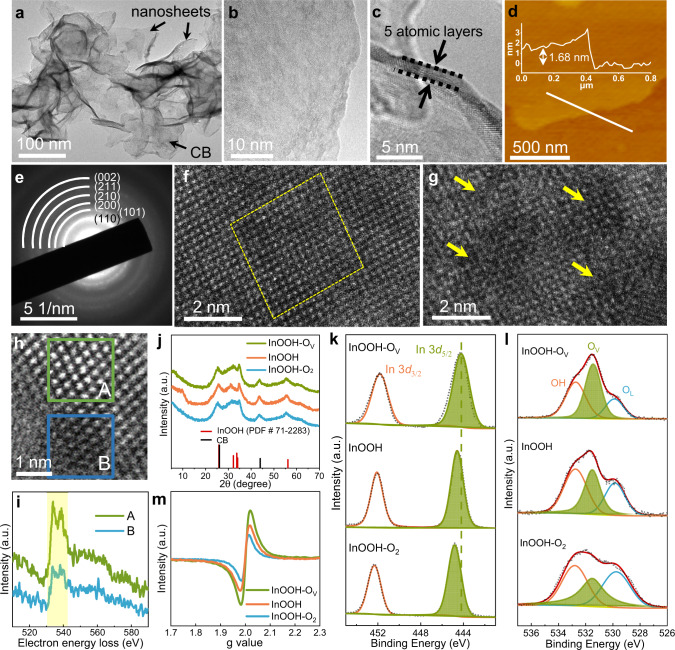
Physical characterization. **a, b** TEM, **c** HR-TEM, and **d** AFM images of InOOH-O_V_. **e** SAED pattern of of InOOH-O_V_. Atomic-resolution HAADF-STEM images of **f** typical intact InOOH nanosheets and **g** InOOH-O_V_. **h** HAADF-STEM image within an individual InOOH-O_V_ sheet and **i** comparison of O K-edge EELS spectra between domain A and domain B. **j** XRD patterns of InOOH-O_2_, InOOH, and InOOH-O_V_ samples. The corresponding HR-XPS spectra of **k** In 3*d* and **l** O 1 *s*. **m** The EPR spectroscopies.

The typical morphology for InOOH nanosheets evenly grown on the surface of conductive carbon black (CB) schematically shown in Fig. 1b can be visualized by scanning electron microscopic (SEM) and transmission electron microscopic (TEM) imaging (Supplementary Fig. 1a–c). For InOOH sample after the Ar or O_2_ plasma treatment (Ar plasma was applied to remove some lattice oxygen atoms in InOOH nanosheet to form more O_V_ sites, while O_2_ plasma was utilized to repair the existed O_V_ sites in the original InOOH nanosheets^19,28,29^), designated as InOOH-O_V_ and InOOH-O_2_, respectively, the nanosheet-like morphologies are well maintained (Fig. 2a, b, and Supplementary Fig. 1). The high resolution- (HR-) TEM image (Fig. 2c) and atomic force microscopy (AFM, Fig. 2d) of InOOH-O_V_ shows ultrathin nanosheets composed of about five atomic layers with an overall thickness of ca. 1.68 nm, drawing the interlayer spacing of ca. 0.34 nm, corresponding to the lattice spacing of InOOH (110) (JPCDS No. 71-2283). The selected area electron diffraction (SAED, Fig. 2e) shows a distinct pattern characteristic of the InOOH crystal with dominance lattice plane (110). The elemental mappings of InOOH-O_V_ show homogenous element distributions for C, O, and In over the sample (Supplementary Figs. 3 and 4), indicating a uniform coverage of InOOH nanosheets on the CB, which could benefit the exposure of active sites and conductivity for the prepared catalysts. The high angle annular dark field scanning transmission electron microscopy (HAADF-STEM) image of a typical intact InOOH nanosheet displays regular periodic alignment of lattice atoms (Fig. 2f); while for a typical InOOH-O_V_ nanosheet, the alignment periodicity of the surface lattice atoms is disrupted with many disordered domains (highlighted by the yellow arrows, Fig. 2g). The observed lattice distorsions indicate the formation of O_V_ sites^30,31^. In addition, the atomic-resolution electron energy-loss spectroscopy (EELS) spectra of O K-edge were acquired within individual sheet but different domains to reflect local chemical states of O element (Fig. 2h, i). The O K-edge spectra are compared between domain A with intact lattice arrangement and domain B with lattice distortion, and the relatively lower peak intensity at 532 eV for the domain B than that of the domain A means the loss of neighboring oxygen coordination, demonstrating the existence of O_V_ sites^32,33^.

X-ray diffraction (XRD) patterns of InOOH, InOOH-O_V_, and InOOH-O_2_ show similar diffraction peaks, characteristic of InOOH at ca. 26.0, 32.2, 33.7, 34.1, and 56.2°, and CB at ca. 26.0 and 44.3° (Fig. 2j, Supplementary Fig. 2), indicating that the plasma treatments did not change the phase structure of InOOH. The surface electronic states of the InOOH, InOOH-O_V_ and InOOH-O_2_ samples were further investigated by X-ray photoelectron spectroscopy (XPS). The survey spectra present similar signals of C, In, and O elements for all three samples (Supplementary Fig. 5, Table S1). Figure 2k, l shows the deconvolved high-resolution XPS (HR-XPS) spectra of In 3d and O 1 *s*, respectively. The peak of In 3*d*_5/2_ at around 444.2 eV for InOOH shifts to lower binding energy after treatment of Ar plasma, but to the higher binding energy after treatment of O_2_ plasma (Fig. 2k), demonstrating the lowest valance state of indium in InOOH-O_V_ (highest valance state of indium in InOOH-O_2_) among the three samples^34,35^. The XPS results for O 1 *s* (Fig. 2i) can be deconvoluted into three peaks located at 529.8, 531.5, and 532.8 eV, attributable to oxygen lattice (O_L_), O_V_, and OH derived from adsorbed water, respectively^36–38^. The relative proportions for the three oxygen species based on the integrated areas are displayed in Table S2. The InOOH sample possesses a higher proportion of the surface O_V_ than O_L_, suggesting that the two-dimensional nanosheet morphology with the high exposed surface is susceptible to the removal of lattice oxygen atoms, thus introducing the O_V_ sites^39^. The proportion of O_V_ is raised to a high value of 40.9% in InOOH-O_V_ with a concomitant decrease in the O_L_ proportion to 19.9%, indicating the removal of lattice oxygen atoms by Ar plasma (Table S2), as also evidenced by the lattice distortion shown in the HAADF-STEM image (Fig. 2g). In contrast, the proportion of O_V_ decreased to 29.0% in InOOH-O_2_ (O_L_ proportion is 34.0%), indicating that O_2_ plasma treatment could repair the surface defects.

There is no obvious change in the OH proportion attributed to adsorbed water among the three samples, which has little effect on electrochemical reactivity (vide infra). The variation of O_V_ induced by plasma treatment is further confirmed by electron paramagnetic resonance (EPR) measurements (Fig. 2m). The signal at g = 2.0035 is attributed to electrons trapped in O_V_^40,41^, and the signal intensity increased in the order of InOOH-O_2_ < InOOH <InOOH-O_V_, which, consistent with the above-mentioned XPS results, shows the highest content of O_V_ for InOOH-O_V_.

**Fig. 3:**
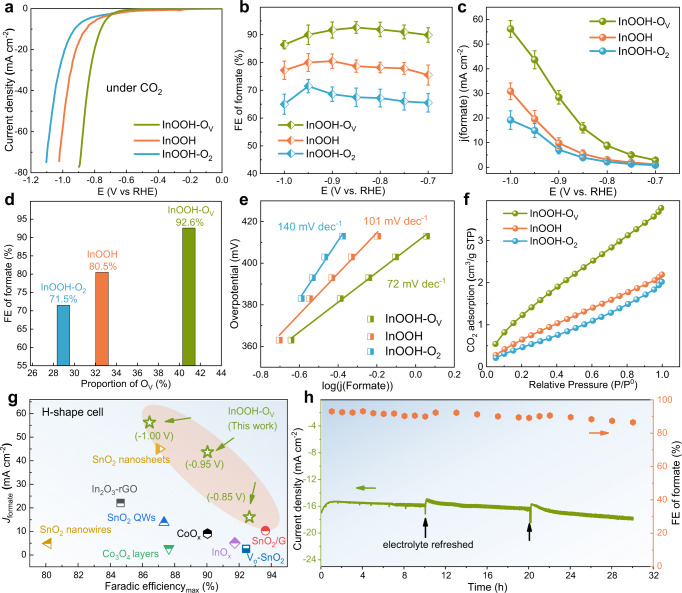
Electrochemical performances for CO_2_RR. **a** The LSV curves for InOOH, InOOH-O_2_, and InOOH-O_V_ under CO_2_ atmosphere. **b** FE of formate and **c** j_formate_ for the three samples. **d** The relationship between max FE of formate and proportion of O_V_. **e** Tafel plots and **f** CO_2_ adsorption tests for InOOH-O_2_, InOOH, and InOOH-O_V_. **g** The comparison of InOOH-O_V_ in formate production efficiency with other reported catalysts using H-shape cell. **h** The stability test of InOOH-O_V_ for 30 h at −0.85 V. The error bars represent the standard deviations for three independent tests under the same conditions.

To evaluate the electrochemical CO_2_RR performance for InOOH, InOOH-O_V_, and InOOH-O_2_ (all the samples for electrochemical tests contain CB as support unless otherwise specified), we used a three-electrode setup with 0.1 M KHCO_3_ as the electrolyte. These samples showed linear scanning voltammetry (LSV) curves with much higher current densities in CO_2_-saturated electrolyte than those recorded in Ar-saturated electrolyte (Supplementary Fig. 6), indicating the occurrence of CO_2_RR. Moreover, the electrolysis current densities for CO_2_RR increased in the order from InOOH-O_2_, InOOH, to InOOH-O_V_, as is the corresponding positive shift in the onset potentials (Fig. 3a), suggesting that the O_V_ contents have considerable influence on the cathodic reaction activity. We further used the potentiostatic method to explore the products for the calculation of the corresponding FE, and online gas chromatography (GC) and hydrogen nuclear magnetic spectra (^1^H-NMR) for the detection of the gas/liquid products (Supplementary Fig. 7), respectively. The products of H_2_, CO, and formate were detected, and the FE towards formate for all samples showed a volcanic trend over the range of -0.70 and -1.00 V, where InOOH and InOOH-O_2_ electrodes owned the maximum FE of 80.5 and 71.5% at -0.90 and -0.95 V, respectively, for formate, while the InOOH-O_V_ electrode achieved the highest FE up to 92.6 % at −0.85 V (Fig. 3b and Supplementary Fig. 8). The carbon source of the obtained formate was demonstrated to be the CO_2_ gas by the fact that almost no CO_2_RR product is probed using CB, itself, as catalyst (Supplementary Fig. 9) and the detailed quantified results of carbon-isotope (^13^CO_2_) experiment using InOOH-O_V_ electrodes without and with CB by ^1^H-NMR and gas chromatography-mass spectrometry (GC-MS) technique (see details in Supplementary Fig. 10). Notably, InOOH-O_V_ with the highest O_V_ content showed the highest selectivity for formate production among the three samples over the applied potentials whereas InOOH-O_2_ exhibited the worst performance. The good performance of InOOH-O_V_ was also reflected by the highest j_formate_ (Fig. 3c), reaching 56.2 mA cm^−2^ at −1.00 V, 43.7 mA cm^−2^ at −0.95 V, and 16.0 mA cm^−2^ at −0.85 V. The positive relationship between the proportion of O_V_ and FE of formate among InOOH, InOOH-O_2_, and InOOH-O_V_ with a similar morphology and mass loading indicates that the enhanced formate production for InOOH-O_V_ is attributable to an increased number of active sites associated with O_V_ (Fig. 3d). The surface OH contents of these three samples varied slightly, but no correlation with the FE of formate was observed (Supplementary Fig. 11), indicating that OH hardly affects the catalytic activity for CO_2_RR.

The effect of electrochemical surface area (ECSA) on the catalytic activity was further investigated through the cyclic voltammetry (CV) scanning curves under the nonfaradaic potentials (Supplementary Fig. 12). The ECSA of InOOH-O_V_, InOOH-O_2_, and InOOH were determined to be 73.3, 83.3, and 60.0 cm^–2^, respectively. The total electrolysis current densities from LSV curves and j_formate_ of the three samples were normalized by the ECSA (Supplementary Fig. 13), which are still proportional to the O_V_ contents, indicating, once again, that the O_V_ plays an important role in determining the efficiency of formate production from CO_2_RR. To investigate the influence of O_V_ on the electron-proton transfer kinetics during CO_2_RR, Tafel plots were presented in Fig. 3e. The Tafel slope of 72 mV dec^−1^ for InOOH-O_V_ is smaller than that of InOOH (101 mV dec^−1^) and InOOH-O_2_ (140 mV dec^−1^), suggesting the most efficient kinetics of InOOH-O_V_ towards HCOO^−^ formation. With the increase of O_V_ content, the value of Tafel slope decreased (even less than 118 mV dec^−1^), indicating a fast electron transfer, and significantly accelerated CO_2_ adsorption and activation processes^42,43^. The positive effect of O_V_ on CO_2_ adsorption process is further confirmed by the CO_2_ isothermal adsorption tests (Fig. 3f) - InOOH-O_V_ exhibited the largest CO_2_ adsorption capacity while InOOH-O_2_ showed the smallest value among the three samples.

Furthermore, the electrochemical impedance spectra (EIS) were tested under CO_2_-saturated electrolyte (Supplementary Fig. 14), and the charge transfer impedance decreased from InOOH-O_2_ through InOOH to InOOH-O_V_, confirming the facilitated Faradaic process due to the increase of O_V_ content^34,35^. Thus, the highest content of O_V_ endows InOOH-O_V_ with the greatest CO_2_ adsorption capability and the fastest CO_2_ activation process, leading to the highest efficiency for formate production. Notably, the excellent catalytic activity observed for InOOH-O_V_ is superior to all the reported In, Co, Cu, and Sn based metal oxide catalysts for CO_2_RR to formate in H-shape cell (Fig. 3g and Table S3). We have also conducted the durability test for InOOH-O_V_ at a given potential of −0.85 V (Fig. 3h), and found the continued stable electrolysis for 30 h with the FE of formate maintaining over 86.5% (electrolyte was refreshed every 10 h). Although there is a tiny part of In metal, the XRD pattern of InOOH-O_V_ electrode after test still exhibites the typical diffraction peaks of InOOH crystal and the proportion of O_V_ still dominated the O species from XPS analysis (Supplementary Fig. 15), which accounts for the good durability of InOOH-O_V_ electrode for CO_2_RR to formate. In sharp contrast, InOOH and InOOH-O_2_ electrodes can only tolerate a period of electrolysis time for 12 h and 6 h at −0.85 V with FE of formate drop to 66.2% and 50.2%, respectively (Supplementary Fig. 15). Due to their weak stability towards CO_2_RR, InOOH and InOOH-O_2_ electrodes showed an additional crystalline phase of In metal generated in InOOH and InOOH-O_2_ electrodes after test, as evidenced in XRD patterns (Supplementary Fig. 15c).

**Fig. 4:**
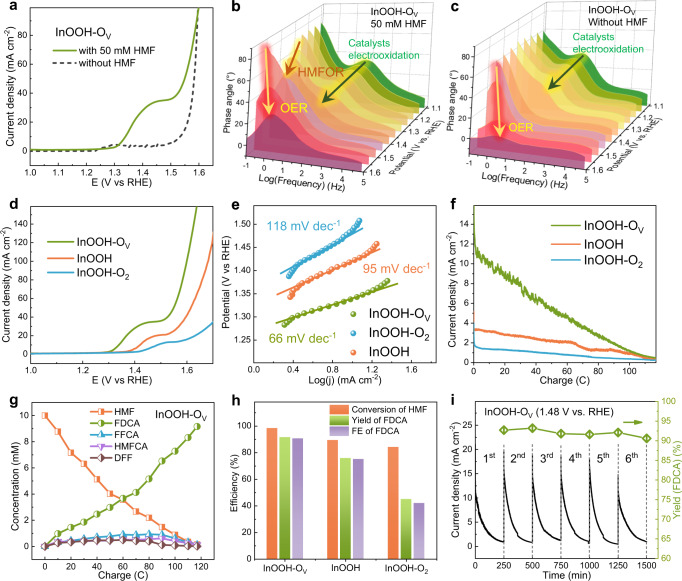
Electrochemical performances for HMFOR. **a** The LSV curves of InOOH-O_V_ in the electrolyte with and without HMF. **b**, **c** Bode phase plots of InOOH-O_V_ in the electrolytes with and without HMF. **d** The LSV curves of InOOH-O_V_, InOOH, and InOOH-O_2_ in 1 M KOH solution with 10 mM HMF at 5 mV s^−1^. **e** The corresponding Tafel plots. **f** The current densities variation over electrolysis charge on the three samples. **g** The concentration variation over electrolysis charge for InOOH-O_V_. **h** The comparison of HMF conversion, FDCA yield, and FE on three samples. **i** The stability test of HMFOR for InOOH-O_V_.

For the anodic HMFOR, InOOH, InOOH-O_2_, and InOOH-O_V_ coated onto a nickel foam (NF) were used for electrocatalytic performance evaluation in a three-electrode setup (see details from the supporting information). LSV curves were measured with or without 50 mM HMF in 1 M KOH solution (pH = 14) at a scan rate of 5 mV s^−1^. As can be seen in Fig. 4a, InOOH-O_V_ exhibited a lower onset potential of 1.30 V for oxidation of HMF (50 mM in 1 M KOH) than that without HMF (1.50 V, for OER only). The low required overpotential indicates the good electrocatalytic activity of InOOH-O_V_ for HMFOR – outperformed all the recently reported catalysts (Table S4). To monitor the oxidation reactions under the applied potentials, the in situ EIS tests for InOOH-O_V_ over a potential gradient from 1.1 to 1.6 V were carried out and the corresponding Bode phase plots were presented in Fig. 4b, c. As can be seen, three peaks could be identified (Fig. 4b). The peak at a frequency above 10^1^ Hz is arising from the normal phenomenon of catalyst electrooxidation^44^, and the other two peaks in the frequency range of 10^−1^ to 10^1^ Hz represent the oxidation reactions at the heterogenous interface; i.e., the peaks for HMFOR and OER at 1.30 and 1.50 V, respectively^19,44–46^, illustrating the preferential potential range for HMFOR is from 1.30 to 1.50 V, which is well consistent with the results from LSV curves (Fig. 4a). However, the in-situ EIS test for InOOH-O_V_ without adding HMF in Fig. 4c only showed two peaks attributed to the catalyst electrooxidation and OER, respectively, further confirming the great promise of InOOH-O_V_ for HMFOR. For InOOH and InOOH-O_2_, the in-situ EIS tests also exhibited the specific peaks for HMFOR (Supplementary Fig. 16). In the presence of 50 mM HMF, the onset potentials for HMFOR are 1.30, 1.37, and 1.41 V for InOOH-O_V_, InOOH, and InOOH-O_2_, respectively. The potential needed to attain current density of 10 mA cm^−2^ is 1.34 V for InOOH-O_V_ electrode, much lower than 1.42 V for InOOH and 1.49 V for InOOH-O_2_ (Fig. 4d and Supplementary Fig. 17). The overpotentials for HMFOR are sequentially lowered from InOOH-O_2_, through InOOH to InOOH-O_V_, suggesting the promoted catalytic activity with the increased contents of O_V_. Moreover, InOOH-O_V_ displayed the lowest Tafel slope (66 mV dec^−1^), compared with InOOH (95 mV dec^−1^) and InOOH-O_2_ (118 mV dec^−1^), indicating that the most accelerated kinetics for HMFOR on the catalyst with the highest content of O_V_ (Fig. 4e). On the other hand, both of the LSV curves from the substrate (NF) and Ar plasma-treated CB on NF substrate showed negligible catalytic activity for HMFOR (Supplementary Fig. 18). Once again, the intrinsic activity for HMFOR is correlated to O_V_ on InOOH-O_V_.

To determine the products of HMFOR, the electrolysis under a constant potential of 1.48 V were employed for InOOH-O_V_, InOOH, and InOOH-O_2_ in 1.0 M KOH (20 mL) electrolyte with 10 mM HMF. It is found that the current densities decreased gradually with the electrolysis charge accumulation (Fig. 4f), most likely due to the consumption of HMF reactants. Notably, the highest current density is observed for InOOH-O_V_, indicating again its highest catalytic activity.

During the electrolysis, the electrolyte was extracted out for high performance liquid chromatography (HPLC) tests to quantify the products based on the calibration curves (Supplementary Fig. 19). The conversion process from HMF to FDCA involves asynchronous oxidation steps of hydroxyl and aldehyde groups, leading to two possible reaction pathways and five chemicals needed to be detected (Supplementary Fig. 20)^14^. The path (I) goes through 5-hydroxymethyl-2-furancarboxylic acid (HMFCA), where the aldehyde group of HMF is oxidized into carboxyl firstly, while in path (II), the hydroxyl in HMF is preferentially oxidized to an aldehyde group, forming 2,5-diformylfuran (DFF). The two paths converge on formyl-2-furancarboxylic acid (FFCA), then lead to the fully oxidized FDCA (Supplementary Fig. 20). From the product quantification results for the three samples (Fig. 4g and Supplementary Figs. 21 and 22), the conversion of HMF approached 98.5% for InOOH-O_V_, demonstrating the high efficiency of InOOH-O_V_ to catalyze HMFOR; while the corresponding values for InOOH and InOOH-O_2_ are only ca. 89.4 and 84.3%, respectively (Fig. 4h and Supplementary Fig. 23). The observed different HMF conversion efficiencies for the three samples could also be directly reflected by the different solution colors after the electrolysis (Supplementary Fig. 24)^14^. Along with the consumption of HMF, the final product of FDCA is gradually accumulated with a FDCA yield of 91.6, 75.9, and 45.0% for InOOH-O_V_, InOOH, and InOOH-O_2_, respectively, at the electrolysis charge accumulation to 117 C (Fig. 4h and Supplementary Fig. 23), indicating again the favorable influence of O_V_ on HMFOR. As such, InOOH-O_V_ presents the highest FE of 90.7% for FDCA (Fig. 4h), demonstrating the high reaction selectivity for the FDCA formation.

The concentration variations of the three intermediates, i.e., HMFCA, DFF, and FFCA, were also investigated (Supplementary Fig. 25) to gain insights in the promotion of HMF conversion and FDCA yield by O_V_. As the electrolysis progressed, InOOH-O_V_ shows the lowest concentration of HMFCA but the highest concentration of FFCA among the three samples, while InOOH-O_2_ presented the opposite results (Supplementary Fig. 25a, b). These results suggest the accelerated transformation of HMFCA to FFCA by the increased content of O_V_, corresponding to the enhanced oxidation of hydroxyl into aldehyde group. Likewise, the introduction of O_V_ also contributed to the conversion of HMF to DFF via pathway (II) as InOOH-O_V_ generated the highest concentration of DFF during the electrolysis (Supplementary Fig. 25c). Therefore, during the HMFOR, the high content of O_V_ on the catalyst can facilitate the oxidation of hydroxyl into aldehyde group, including the steps from HMF to DFF and from HMFCA to FFCA (Supplementary Fig. 20), leading to the significantly improved HMF conversion, and thus a high yield of FDCA. Furthermore, six sequential electrolysis batches of HMFOR on InOOH-O_V_ at 1.48 V presented similar current curves with FDCA yields and FE maintaining over 90.0% (Fig. 4i and Supplementary Fig. 26) while InOOH-O_V_ electrode after test still shows the main crystalline phase of InOOH and the dominated proportion of O_V_ sites among O species (Supplementary Fig. 27), revealing a long-term stability for InOOH-O_V_ towards HMFOR. In contrast, the InOOH and InOOH-O_2_ electrodes after six electrolysis cycles of HMFOR at 1.48 V exhibited much descended FDCA yield to 50.6% and 14.2%, respectively, with a crystalline phase of In(OH)_3_ generated. These results demonstrate the high activity and stability of the active sites in InOOH-O_V_ (Supplementary Fig. 27).

**Fig. 5:**
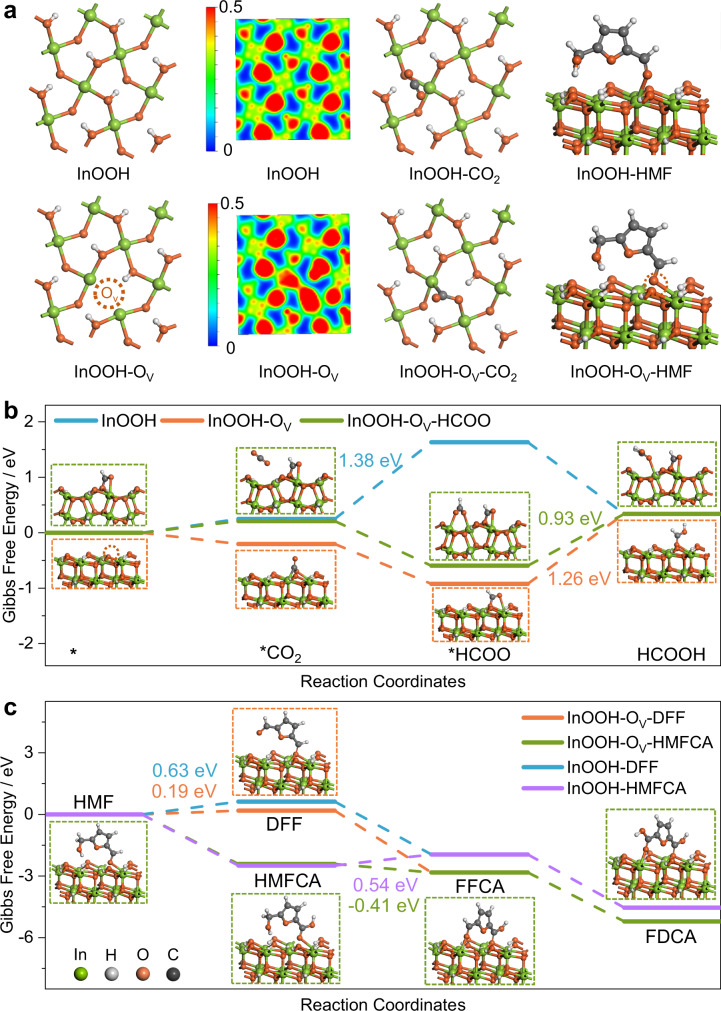
Theoretical calculations. **a** The diagrams (top view) of built models of InOOH and InOOH-O_V_ and their corresponding ELF, and the adsorption configurations of CO_2_ and HMF. **b** Free energy diagram for CO_2_RR on InOOH, InOOH-O_V_, and InOOH-O_V_-HCOO with the intermediate adsorption configurations for InOOH-O_V_ and InOOH-O_V_-HCOO. **c** Free energy diagram for HMFOR on InOOH and InOOH-O_V_ with the adsorption configurations of five products for InOOH-O_V_. Color code: green, In; grey, C; red, O; white, H.

Density functional theory (DFT) calculations were performed to understand the catalytic mechanism of the surface O_V_ on InOOH nanosheets for CO_2_RR and HMFOR, respectively. Two models were established (Fig. 5a), including the intact InOOH lattice plane (110) with a slab of five atomic layers based on the HRTEM and atomic force microscopy (AFM) analysis (Fig. 2c, d), and the same plane with one surface oxygen atom removed to form O_V_ (InOOH-O_V_). Herein, the selection of InOOH lattice plane (110) is based on the comprehensive characterization and analyses of XRD, SAED, HR-TEM and AFM images (Fig. 2c–e, j, vide supra). The electron localization function (ELF) of the two models, reaction intermediates and product adsorption behaviors, and the Gibbs free energy changes along the conversion paths were simulated. As shown in Fig. 5a, upon the introduction of O_V_ onto the InOOH (110) surface, the two In atoms adjacent to the removed O atom show extra electron aggregation (see the ELF plots), and this charge redistribution induces new adsorption configurations of CO_2_ and HMF molecules, distinguished with those on intact InOOH surface (vide infra). The charge redistribution, calculated by the Perdew-Burke-Ernzerhof (PBE) exchange-correlation functional, can be further explained by the partial density of state (PDOS) of the p-orbital on In atoms (Supplementary Fig. 28a, b). The p-orbital of In atom on intact InOOH (110) plane shows no apparent PDOS at Fermi level, indicating the difficult electron transfer between the In atom and reactant molecules, reflecting the pristine electronic properties for main-group p-block metals; while the p-orbital of the adjacent In atom at O_V_ site exhibits PDOS right at Fermi level, which means that the electrons on the highest occupied molecular orbital (HOMO) can be transferred to the lowest unoccupied molecular orbital (LUMO) easily, benefitting the formation of the covalent bond between the adjacent In atom and adsorbate intermediates (Fig. 5a), then facilitating the subsequent electrochemical catalytic reactions. Besides, the hybrid functional HSE06^47^ was used to examine the electron distribution of the InOOH system. The similar PDOS distribution of In atom indicates the negligible error of functional PBE on this system, as shown in Supplementary Fig. 28c, d.

The process of CO_2_RR to formate can be thermodynamically divided into three steps^22,48^: the first step is CO_2_ adsorption and activation to form the intermediate *CO_2_, followed by the formation of HCOO* and its hydrogenation to HCOOH, which finally desorbes from the catalyst surface. For InOOH-O_V_, CO_2_ molecule can be implanted into the O_V_ site through the chemical binding of C and O atoms to the two electron-rich In atoms, facilitating CO_2_ adsorption and activation process (Fig. 5a, b), which is consistent with the Tafel plots (Fig. 2e) and CO_2_ adsorption isotherms (Fig. 2f). While the intact lattice plane of InOOH is not conducive to CO_2_ adsorption (Supplementary Fig. 29), hindering the subsequent electron transfer and protonation processes. The protonation step of CO_2_* with energy differences (ΔG) of 1.38 eV is the potential determining step (PDS) for InOOH. While for InOOH-O_V_, the facilitated CO_2_ activation benefits the next protonation step of *CO_2_ to form HCOO*, making the HCOO* desorption step become the PDS with a lower ΔG of 1.26 eV (Fig. 5b). Interestingly, it was found that the ΔG for HCOO* hydrogenation can be further reduced to 0.93 eV when a second CO_2_ molecule subsequently adsorb onto the InOOH-O_V_ surface with attached HCOO* (InOOH-O_V_-HCOO, Fig. 5b). These results indicate that the introduction of O_V_ onto InOOH (110) surface benefits the CO_2_ adsorption and activation, and changes the step of PDS to HCOO* hydrogenation and reduces the corresponding ΔG to a much lower value for formate production, leading to the high performance of InOOH-O_V_ towards CO_2_RR. We further adopted the revised PBE (RPBE) functional to examine the functional sensitivity for CO_2_RR modeling, and the relative energy trends are similar (Supplementary Fig. 30). In addition, we investigated the major competition product (H_2_, CO) to identify the selectivity of HCOOH. As shown in Supplementary Fig. 31, the energetic favor to *CO_2_ adsorption illustrates higher selectivity of CO_2_RR compared with HER, while the product of *CO_2_ hydrogenation trends to *HCOO rather than *COOH, indicating the higher selectivity of HCOOH.

Furthermore, Gibbs free energy evolutions were studied for the two paths of HMFOR into FDCA (Fig. 5c, Supplementary Figs. 20 and 32). For both the two models for InOOH and InOOH-O_V_, the HMFOR process prefers the path (I) to form HMFCA with a negative Gibbs free energy change, rather than path (II) forming DFF, with an ΔG of 0.63 and 0.19 eV, respectively. In path (I), the PDS for both InOOH and InOOH-O_V_ is the step from HMFCA to FFCA with an ΔG of 0.54 and -0.41 eV, respectively, indicating that the O_V_ site facilitates the oxidation of hydroxyl into aldehyde group. Similar O_V_ effect also works for the step from HMF to DFF (the PDS for path II) with a much lower ΔG of 0.19 eV for InOOH-O_V_ than that of 0.63 eV for InOOH, indicating the higher reaction activity for hydroxyl oxidation on InOOH-O_V_. Therefore, both the DFT simulations and the experimental results confirm that the O_V_ sites can facilitate the oxidation of hydroxyl into aldehyde group during HMFOR, and thus facilitating the FDCA yield.

**Fig. 6:**
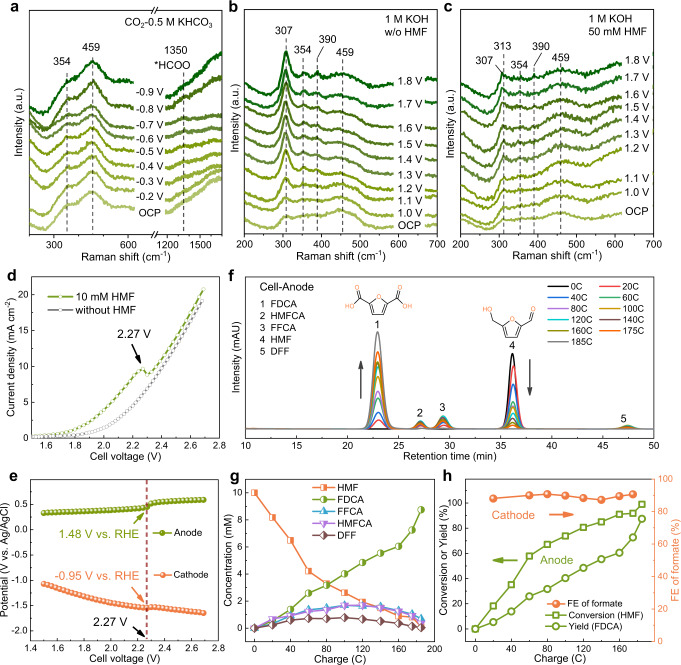
Operando Raman spectra and electrochemical performances for integrated cell. The operando Raman spectra of InOOH-O_V_ in the electrolyte of **a** CO_2_ saturated 0.5 M KHCO_3_, **b** 1 M KOH, and **c** 1 M KOH with 50 mM HMF. **d** The LSV curves of the integrated cell in the electrolytes with and without HMF. **e** The bias potentials for cathode and anode. **f** The HPLC signals for anodic products. **g** The variation of anodic products concentration. **h** The HMF conversion, FDCA yield, and FE of formate in the integrated cell.

To monitor the catalyst structure dynamics during the CO_2_RR and HMFOR processes, the operando Raman spectra were collected through a custom-built H-shape electrolysis cell with an optical quartz window (Supplementary Fig. 33). As shown in Fig. 6a, the potential-dependent in-situ Raman spectroscopy of InOOH-O_V_ for CO_2_RR was acquired in the range of 200 and 1700 cm^–1^ in CO_2_-saturalted 0.5 M KHCO_3_ electrolyte. At open circuit potential (OCP), two typical bands were recognized at 354 and 459 cm^–1^, which can be assigned to the In-O vibrations in InOOH^49^. When the applied potentials were regulated from OCP to -0.4 V, an additional Raman band emerged at 1350 cm^–1^, which can be attributed to the O-C-O symmetric stretch mode of the key intermediate *HCOO during formate formation^50^. The peak intensity corresponding to *HCOO gradually enhanced with the potential moving negatively, reached the maximum at –0.8 V (accordant to the evolution trend of FE of formate over InOOH-O_V_, Fig. 2b), demonstrating the generation of formate. It is worth noting that no obvious change is observed on the two In-O bands at 352 and 459 cm^–1^ with the varied potentials, that is, the oxidation state of In elements in InOOH-O_V_ is well-maintained during CO_2_RR. This phenomenon is distinguished from other metal oxide catalysts (e.g., SnO_2_^51^ and InOOH^52^ etc.), which will be fully/partially reduced to metal with zero/lower valence, being the real active sites for CO_2_RR to formate. In this context, O_V_ sites keep the Indium elements at low oxidation valance (Fig. 2k), which is hard to be further reduced during CO_2_RR, benefiting the controllability and durability of InOOH-O_V_.

On the anodic side, the potential-dependent in-situ Raman spectroscopy of InOOH-O_V_ was performed in 1 M KOH solution (Fig. 6b). Except the two peaks attributed to In-O bands at 354 and 459 cm^–1^, an emerging peak located at 307 cm^–1^ appeares with its peak intensity gradually enhances with increasing the potential. In addition, another peak at 390 cm^–1^ emerged at 1.2 V. Two typical peaks at 307 and 390 cm^–1^ could be attributed to the In-OH stretching vibration modes^53^, which are believed to derive from the adsorption and concentration of OH ions under the basic environment for the following OER process^54^. When 50 mM HMF was added in 1 M KOH electrolyte (Fig. 6c), an additional peak is observed at 313 cm^–1^ at OCP, presenting a blue-shift by 6 cm^–1^ from 307 cm^–1^, arising from the competition of HMF molecules with the OH^−^ in the solution. The preference of O_V_ sites to being occupied by HMF molecules could be corroborated by the OCP measurements with different HMF concentrations of 10, 20, and 50 mM (Supplementary Fig. 34). The peak at 390 cm^–1^ did not appear until 1.5 V, showing 300 mV later that without HMF adding. The band at 313 cm^–1^ red-shifted back to 307 cm^–1^ above 1.5 V, indicating that OH ions overwhelm the superiority over HMF molecules to adsorb at the O_V_ sites, and thus OER becomes the dominant reaction, which is accordant to the result from LSVs in Fig. 4a. The HMFOR process is also confirmed by the operando Raman spectroscopies obtained in the range of 1300 and 1700 cm^–1^ (Supplementary Fig. 35). The Raman band at 1514 cm^–1^ appearing between 1.3 and 1.5 V in 1 M KOH solution with 50 mM HMF is assigned to a C = C stretching mode for FDCA formation^55^, which is not observed in the case without HMF.

The remarkable bifunctional activity of InOOH-O_V_ towards CO_2_RR and HMFOR holds great promise in developing a two-electrode integrated system, where the anodic biomass valorization (generating FDCA) and cathodic CO_2_ conversion to formate production are simultaneously achieved (Fig. 1a) with attractive system-level performance. Herein, the gas-tight two-compartment electrolysis cell was assembled with InOOH-O_V_ on NF as anode and InOOH-O_V_ on carbon paper as cathode, respectively. The anodic chamber contained 1 M KOH solution containing 10 mM HMF (pH = 14), while the cathodic chamber was filled with 0.1 M KHCO_3_ solution bubbled with CO_2_ gas flow (pH = 6.8), and two chambers were separated by a BPM (Fig. 1a). A typical BPM consists of laminated films of anion-exchange layer (AEL) and cation-exchange layer (CEL) with a bipolar interfacial layer (IL) formed between that allows selective diffusion of protons and hydroxide anions towards the negative and positive electrode, respectively (Supplementary Fig. 36). The CEL-AEL interface maintains the generated pH gradient across the BPM during electrolysis due to the ions permselectivity of each respective film and the electrokinetics at the CEL-AEL interface under forward and reverse biases^56^, which affords the coupling of alkaline HMFOR and neutral CO_2_RR in separated electrode compartments.

The LSV curves are compared within the potential range of 1.5 ~ 2.7 V from the electrolytes with and without 10 mM HMF, and the current densities are distinctly higher when HMF is added, indicating the strong promotion of cell performance with HMFOR replacing OER at the anode (Fig. 6d). With the increase in the cell voltage, a peak appears at 2.27 V for LSV curve, which could be explained by the demarcation point where the OER outcompets the HMFOR. The bias potentials for anode oxidation and cathode reduction were monitored by the LSV test. When the cell voltage reaches 2.27 V, the anodic and cathodic bias potentials are located at 1.48 and −0.95 V (Fig. 6e), corresponding to the optimal potentials for HMFOR and CO_2_RR, respectively (vide supra), which demonstrats the integration compatibility of the reaction couple based on the bifunctional InOOH-O_V_ catalyst. 2.27 V is selected as the constant potential for cell electrolysis as both the bias potentials for anode and cathode swung positively due to the predominating OER, when the applied potential becomes more positive. During the whole process, the cathodic CO_2_RR products and anodic HMFOR products are monitored simultaneously (Supplementary Figs. 7 and 19).

With the electrolysis charge accumulating to 185 C, the HMF conversion rate achieves as high as 99.0%, the corresponding FDCA yield reaches to 87.5%, with the FE of formate remained over 90.0% all the time (Fig. 6f–h). The combined electron efficiency of the intergrated cell is determined to reach 172.1%, nearly double those of the independent HMFOR and CO_2_RR, respectively (Supplementary Fig. 37), demonstrating the great advantage of intergrated cell for reducing electricity consumption. This performance has clearly demonstrated the successful integration of HMFOR and CO_2_RR within an integrated electrolysis cell and the great potential for using InOOH-O_V_ as a bifunctional catalyst to promote the electrolysis system, opening a pathway for other prospective applications.

In this work, indium oxyhydroxide nanosheets with different contents of oxygen vacancies (O_V_) were tuned via a plasma treating method, and the sample rich in O_V_ (InOOH-O_V_) was demonstrated as a superior bifunctional catalyst for electrochemical CO_2_RR to value-added formate with maximum FE and current density of 92.6% and 56.2 mA cm^−2^, respectively, along with biomass valorization process of HMFOR to FDCA with a yield of 91.6%. These results are among the top records for both the conversion of CO_2_ and HMF. The decisive role of O_V_ in the bifunctional activities and the intrinsic catalytic mechanisms were revealed by DFT calculations and operando Raman spectra, indicating that the charge redistribution at the O_V_ sites affected the adsorption behaviors of reaction intermediates to ensure the high catalytic activities. The realized activities of InOOH nanosheets in this study provide a practicable approach to developing main-group p-block metal oxides as efficient bi/multi-functional electrocatalysts. More importantly, the successful integration of CO_2_RR and HMFOR with InOOH-O_V_ as a bifunctional catalyst and BPM to separate the electrolyte with asymmetric pH values provides a valuable reference to integrate electrolysis processes for biomass valorization and CO_2_ conversion, opening a pathway for other prospective applications for the generation of commodity chemicals simultaneously on both electrodes in one electrolyzer.

## Methods

### Preparation of InOOH, InOOH-O_V_, and InOOH-O_2_

Typically, 270 mg of In(NO_3_)_3_·4H_2_O and 2 g of urea were added into 60 mL of ethanol and kept stirring until completely dissolved. Then, 65 mg of CB (commercial XC-72R) was added into the solution to form a well-dispersed mixture by ultrasound treatment for 30 min. A solvothermal process was applied to the mixture at 90 °C for 12 h in a Teflon-lined autoclave with a volume of 100 mL. Subsequently, the resulting sample was filtered, washed with plenty of ethanol and ultrapure water until neutral, and then dried at 60 °C for 12 h under vacuum. The obtained sample was labeled as InOOH. To adjust the surface content of O_V_, InOOH was treated by Ar and O_2_ plasma for 120 s (100 W, 20 pa), respectively, the resultant samples were labeled as InOOH-O_V_ and InOOH-O_2_, respectively (the sample of InOOH-O_V_ without CB for operando Raman spectra acquisition was also prepared).

### Electrochemical tests

The as-obtained catalysts together with 10 wt.% Nafion ionomers were suspended in an isopropanol solution (35 % in water) under ultrasonic operation for ca. 20 min to get the well-dispersed ink. For cathodic CO_2_RR, the ink was coated onto a piece of hydrophobic carbon cloth (CC) at 70 °C to make a gas diffusion electrode, while for anodic HMFOR, the ink was dropped onto a piece of nickel foam (NF) dried by an electric blower. The sample loadings were both fixed at 2 ± 0.05 mg cm^−2^, and the geometric surface area of the working electrode was 1.0 and 2.0 cm^−2^ for the cathode and anode, respectively.

For CO_2_RR, the electrochemical tests were carried out in a gastight H-shaped electrolytic cell with two compartments separated by cation-exchange membrane (Nafion 117). Before the electrochemical tests, each compartment was added 40 mL KHCO_3_ (0.1 M) solution as an electrolyte, followed by being bubbled with ultrapure CO_2_ gas (99.999 %) for at least 30 min to achieve CO_2_ saturation (pH = 6.8). The gas flow rate was finely controlled by an electric mass flow controller (MFC) at 30 ml min^−1^. The electrolysis was conducted under stirring at 400 rpm, with a piece of the platinum plate as the counter electrode and an Ag/AgCl reference electrode. All electrochemical tests were controlled by an electrochemical workstation (CHI760E) and the potentials in this work were converted by the formula E (vs. RHE) = E (vs. Ag/AgCl) + 0.197 V + 0.0591 × pH. The linear scanning voltammetry (LSV) tests were conducted in the range of 0 to -1.1 V vs. RHE at scanning rate of 5 mV s^−1^. The electroreduction of CO_2_ was performed by potentiostatic method, and each applied potential was kept for 30 min. The off-gas from the cathodic compartment was monitored to determine the gas products by an online gas chromatography (Shimadzu GC 2014) equipped with a TCD detector and a FID detector. The electrolyte after test was collected and analyzed with hydrogen nuclear magnetic resonance (^1^H-NMR, Bruker 400 MHz) to determine the liquid products. The electrochemical impedance spectra (EIS) were recorded under optimal reaction potential in the frequency range of 10^5^ ~ 10^−1 ^Hz. The uncompensated solution resistance (R_u_) was compensated for 90% during electrolysis. The current densities were calculated based on the geometric projected electrode area.

For HMFOR tests, the LSV curves were collected in a one-chamber undivided cell in between 1.0 and 1.7 V vs. RHE with scanning rate of 5 mV s^−1^ and the electrolysis at fixed potential was conducted in a H-shaped electrolytic cell with two compartments separated by cation-exchange membrane (Nafion 117). 1 M KOH solution with 50 mM or 10 mM HMF was utilized as an electrolyte. A graphite rod was used as the counter electrode and a Hg/HgO electrode was used as the reference electrode, respectively. All electrochemical tests were controlled by an electrochemical workstation (CHI760E) and the potentials in this part were converted by the formula E (vs. RHE) = E (vs. Hg/HgO) + 0.098 V + 0.0591 × pH. The EIS tests were recorded in the frequency range of 10^5^ ~ 10^−1 ^Hz. The uncompensated solution resistance (R_u_) was compensated for 90% during electrolysis. The current densities were calculated based on the geometric projected electrode area. The concentrations of HMF and oxidized products were examined by high-performance liquid chromatography (HPLC, Thermo U-3000) equipped with a photo-diode array (PDA) detector and a Aminex HPX-87H chromatographic column. The wavelength of PDA detector was set at 265 nm and the column temperature was kept at 50 °C. Sulfuric acid solution (5 mM) was used as the mobile phase at a flow rate of 0.6 mL min^−1^. During the electrolysis, the anodic electrolyte was extracted and diluted 10 times with ultrapure water for HPLC detection. The HMF conversion, FDCA yield, FE of FDCA, and the combined electron efficiency (EE) are calculated according to the following equations.

4 $${{{{{\rm{HMF}}}}}}\,{{{{{\rm{conversion}}}}}}\,(\%)=\frac{n\,({{{{{\rm{HMF}}}}}}\,{{{{{\rm{consumed}}}}}})}{n\,({{{{{\rm{HMF}}}}}}\,{{{{{\rm{initial}}}}}})}\times 100$$

5 $${{{{{\rm{FDCA}}}}}}\,{{{{{\rm{yield}}}}}}\,(\%)=\frac{n\,({{{{{\rm{FDCA}}}}}}\,{{{{{\rm{formed}}}}}})}{n\,({{{{{\rm{HMF}}}}}}\,{{{{{\rm{initial}}}}}})}\times 100$$

6 $${{{{{\rm{FE}}}}}}\,{{{{{\rm{of}}}}}}\,{{{{{\rm{FDCA}}}}}}\,(\%)=\frac{6F\cdot n({{{{{\rm{FDCA}}}}}}\,{{{{{\rm{formed}}}}}})}{Q}\times 100$$

7 $${{{{{\rm{EE}}}}}}\,(\%)=\frac{F(6n\,({{{{{\rm{FDCA}}}}}}\,{{{{{\rm{formed}}}}}})+2n\,({{{{{\rm{HCOOH}}}}}}\,{{{{{\rm{formed}}}}}}))}{Q}\times 100$$

where, *n* is the molar concentration of relative chemicals, F is the Faraday constant as 96485 C mol^−1^, and Q is the electrolysis charge, C.

The integrated electrolysis was conducted in divided two-compartment cell using two-electrodes system. The sample InOOH-O_V_ was coated onto carbon cloth and nickel foam as the cathode and anode, respectively. The cathode was in size of 1 × 2 cm, while the anode was in size of 2 × 2 cm. The cathodic electrolyte was 30 mL CO_2_ saturated 0.1 M KHCO_3_ and the anodic electrolyte was 30 mL Ar saturated 1.0 M KOH with 10 mM HMF. In consideration of the asymmetrical pH between the two chambers, they were separated by a bipolar membrane (BPM). The LSV curves were recorded between 1.5 and 2.7 V with scanning rate of 5 mV s^−1^. All electrochemical tests were controlled by an electrochemical workstation (CHI760E). In order to monitor the bias voltages of two electrode reactions, two Ag/AgCl electrodes were placed near cathode and anode, respectively.

### Physical characterization

X-ray diffraction (XRD) patterns were acquired on a Bruker D8 Advanced X-ray diffractometer. The electron microscopy images for samples were obtained by a field emission transmission electron microscope (TEM, FEI Tecnai G2 20 S Twin microscopy, 300 kV) and a scanning electron microscope (SEM, HitachiS-5200). A Thermo Fisher Scientific ESCALAB 250 was utilized for X-ray photoelectron spectroscopies (XPS). EPR analyses were conducted on a Bruker EMX PLUS. A Micromeritics ASAP 2020 HD88 analyzer was applied for CO_2_ adsorption evaluation. Before measuring for CO_2_ adsorption at 298 K, a degas process at 393 K under vacuum was applied for the InOOH-O_V_, InOOH, and InOOH-O_2_. HAADF was conducted on an aberration-corrected JEM-ARM300F GRAND ARM with an operating voltage of 300 kV (Technical Institute of Physics and Chemistry, the Chinese Academy of Sciences, Beijing). MS spectra were acquired on a GC-MS QP2010 ultra (Shimadzu, Kyoto, Japan).

### In-situ/operando Raman spectroscopy

The operando Raman spectra were collected through a custom-built H-shape electrolysis cell with an optical quartz window (EC-RAIR-H, Beijing Scistar Technology Co. Ltd, as shown in Supplementary Fig. 33), using a 633 nm laser (InVia Reflex). The sample powder was dropped onto a glass carbon electrode as the working electrode, and a platinum wire was used as the counter electrode, with a Ag/AgCl electrode as the reference electrode. For CO_2_RR, the CO_2_-saturated 0.5 M KHCO_3_ solution was pumped into the electrolysis cell as the electrolyte. For HMFOR, 1 M KOH containing 50 mM HMF was used.

### Computational methods

The spin-polarized calculations within the density functional theory (DFT) framework were carried out by the Vienna ab initio simulation package (VASP)^57^. The interaction between the ions and the electrons with the frozen-core approximation was represented by the projector-augmented wave (PAW) method^58^ and the electron exchange-correlation by the generalized gradient approximation (GGA) with the PBE exchange-correlation functional^59^. Revised PBE (RPBE) function was used to examine the functional sensitivity for CO_2_RR. The hybrid functional HSE06^47^ was used to identify the negligible effect of PBE functional on the calculation of the density of state (DOS). A cut-off energy of 400 eV was employed for the plane-wave basis set. The Brillouin-zone integrations were performed using a (2 × 2 × 1) Monkhorst-Pack mesh during the optimization. The iterative process considered was converged when the force on the atom was <0.05 eV Å^−1^ and the energy change was <10^−4 ^eV per atom. Data of the converged calculations are provided as Datasets 1–3 in Supplementary Data.

InOOH(110) surface was modeled with a slab of five atomic layers, in which the bottom three layers were frozen, and a vacuum layer of about 15 Å along the z-axis was built. One surface oxygen atom is removed to establish InOOH with oxygen vacancy (InOOH-O_V_). During geometry optimization, the bottom three layers also were fixed for InOOH-O_V_. The Gibbs free energies (G) at 298.15 K and 1 atm were calculated by:

$$G=H-TS={E}_{DFT}+{E}_{ZPE}+{\int }_{0}^{298.15\,K}{C}_{V}dT-TS$$

where E_DFT_ is the total energy obtained from DFT optimization, E_ZPE_ is the zero-point vibrational energy using the harmonic approximation^60^, C_V_ is the heat capacity, T is the kelvin temperature, and S is the entropy. The entropies of gas molecules were taken from NIST database. The free energy of O_2_ was extracted from the O_2_ + H_2_ → 2H_2_O (l) reaction because the high-spin ground state of the O_2_ molecule is poorly described in DFT calculation^61,62^. And the free energy of liquid water was calculated as an ideal gas at 3534 Pa, which corresponds to the vapor pressure of water at which point the chemical potential of liquid and vapor phases are equal^63^. Similarly, formic acid was calculated as an ideal gas at 2.0 Pa, which corresponds to an aqueous-phase activity of 0.01^63^. The computational hydrogen electrode (CHE) model^64^ was used to calculate the free energy of electrocatalytic CO_2_RR. In this work, the implicit solvent model was considered for the effects of water solvent environment^65^.

## Supplementary information

Supporting Information

Description of Additional Supplementary Files

Dataset 1-3

## Data Availability

The authors declare that the data supporting the findings of this study are available within the article and its Supplementary Information/Source Data file/Supplementary Data. Any additional detail can be requested from the corresponding author (C.H.). Source data are provided with this paper.

## Source data

Source Data
