# Supplementary material for: The role of oxygen-vacancy in bifunctional indium oxyhydroxide catalysts for electrochemical coupling of biomass valorization with CO2 conversion
Source: Nat Commun. 2023 Apr 11;14:2040. doi: 10.1038/s41467-023-37679-3 (PMC10090200; doi:10.1038/s41467-023-37679-3)
Supplement: Supplementary file 1 — Supporting Information [file 41467_2023_37679_MOESM1_ESM.pdf]

Supporting Information

The Role of Oxygen-Vacancy in Bifunctional Indium Oxyhydroxide Catalysts for Electrochemical Coupling of Biomass Valorization with CO_2 Conversion

*Fenghui Ye,[a] Shishi Zhang,[b] Qingqing Cheng,[c] Yongde Long,[a] Dong Liu,[a] Rajib Paul,[d] Yunming Fang,[*a] Yaqiong Su,[*b] Liangti Qu,[e] Liming Dai,[f] Chuangang Hu[*a]*

[a]*State Key Laboratory of Organic-Inorganic Composites, College of Chemical Engineering, Beijing University of Chemical Technology, Beijing 100029, China*

[b]*School of Chemistry, Xi'an Key Laboratory of Sustainable Energy Materials Chemistry, State Key Laboratory of Electrical Insulation and Power Equipment, Xi'an Jiaotong University, Xi'an 710049, China*

[c]*Shanghai Advanced Research Institute, Chinese Academy of Sciences, Shanghai, 201210, China*

[d]*Advanced Materials and Liquid Crystal Institute, Kent State University, Kent, OH 44242, USA*

[e]*Department of Chemistry, Tsinghua University, Beijing 100084, China*

[f]*ARC Centre of Excellence for Carbon Science and Innovation, University of New South Wales, Sydney, NSW 2052, Australia*

** Corresponding author, E-mail address:*

fangym@mail.buct.edu.cn; yqsu1989@xjtu.edu.cn; chuangang.hu@mail.buct.edu.cn

Table of Contents

Characterization of prepared samples S3

CO_2RR performances of InOOH, InOOH-O_V, and InOOH-O_2..S8

HMFOR performances of InOOH, InOOH-O_V, and InOOH-O_2... S18

DFT calculations..S30

Operando Raman tests...S35

Electrochemical performances of integrated cell..S38

Supplementary Tables...S40

References S41

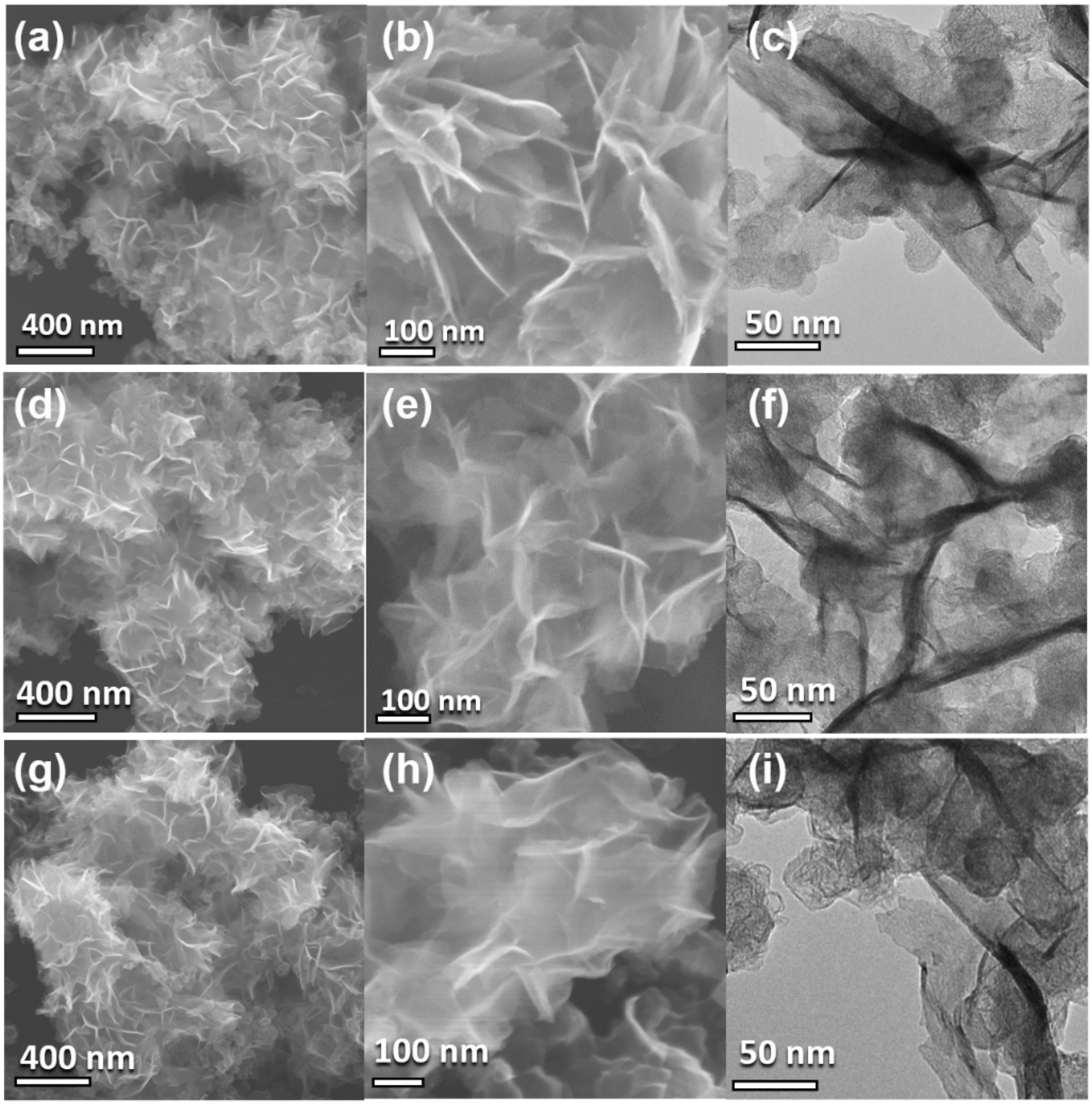

Supplementary Figure 1. The electron microscopy images for three samples. The SEM images (a, b) and TEM image (c) of InOOH. The SEM images (d, e) and TEM image (f) of InOOH-O_V. The SEM images (g,h) and TEM image (i) of InOOH-O_2

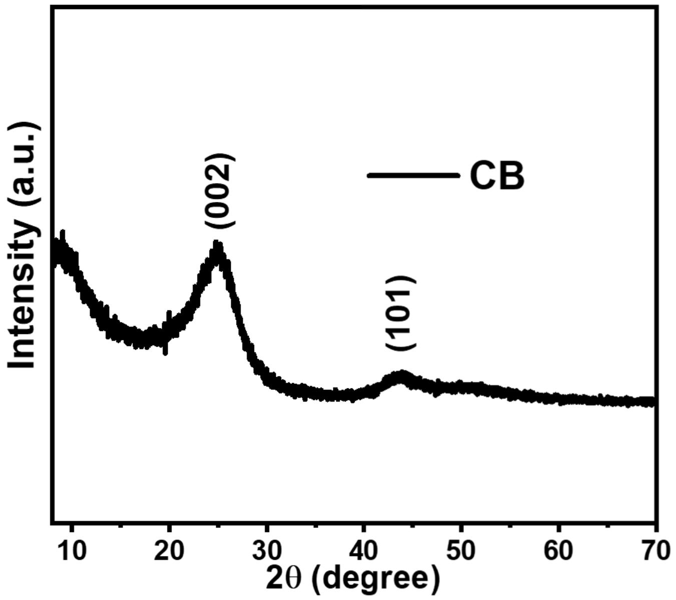

Supplementary Figure 2. XRD pattern of CB. Two characteristic diffraction peaks at 26.0 and 44.3° appeared.

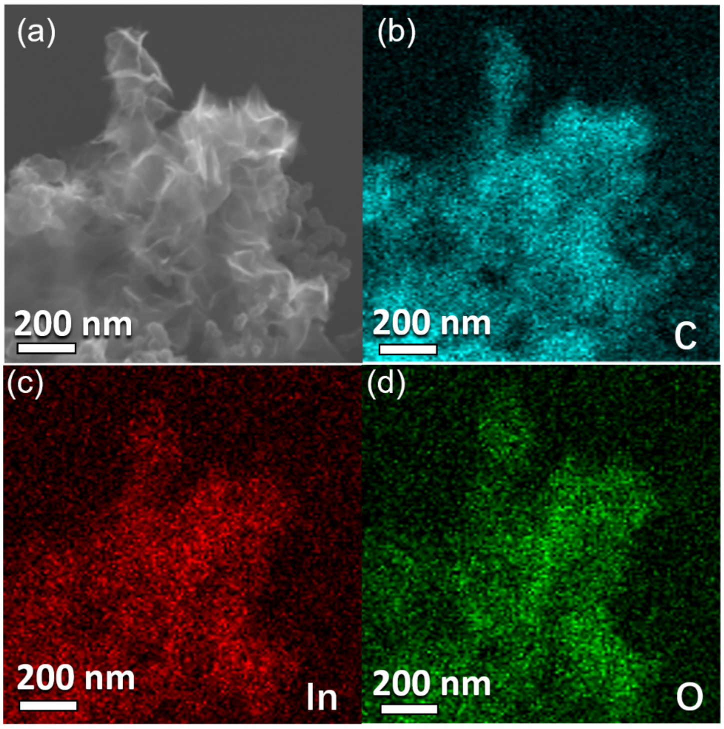

Supplementary Figure 3. Element distribution analysis. (a) SEM image and (b-d) corresponding EDS mapping images of C, In, and O elements for the InOOH nanosheets grew on CB.

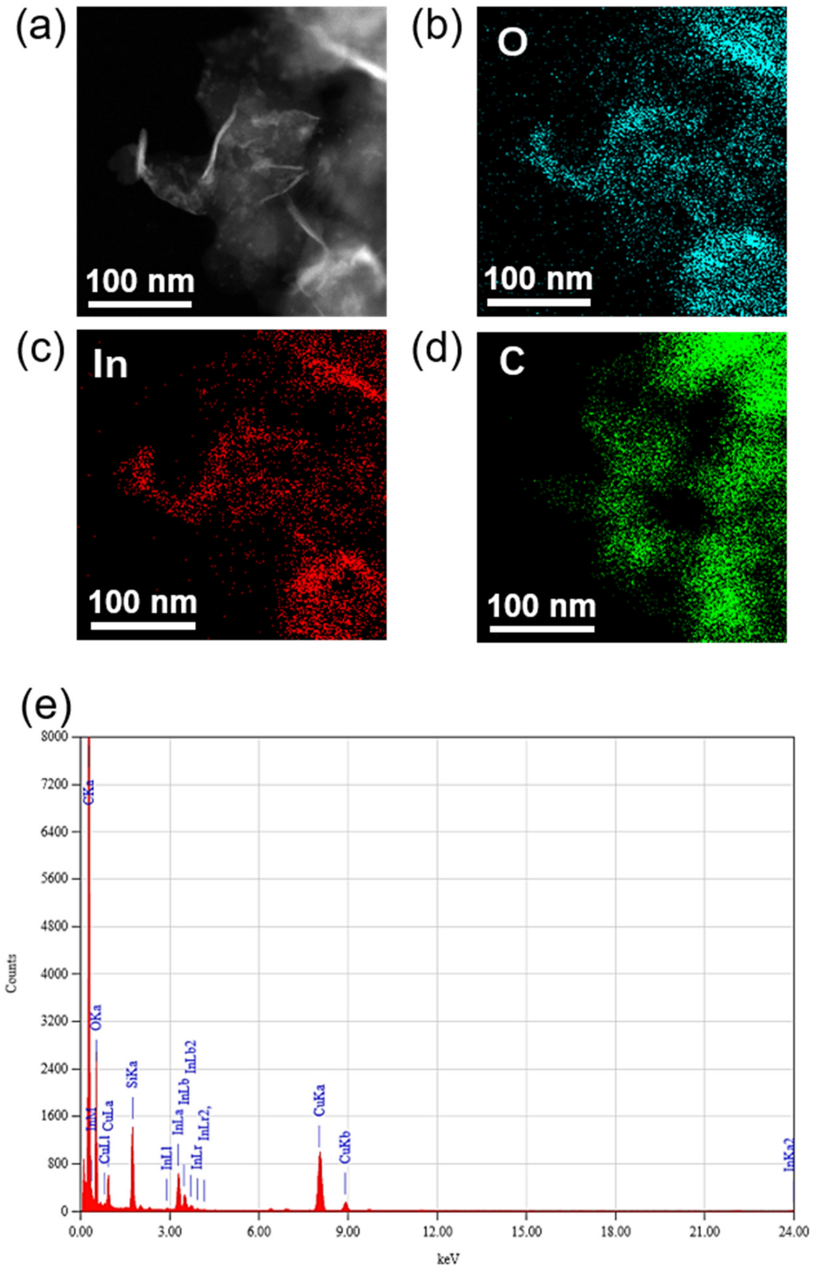

Supplementary Figure 4. Element distribution for InOOH nanosheets. (a) TEM image and (b-d) corresponding EDS mapping images of O, In and C elements of InOOH nanosheets grewed on CB. (e) The extracted EDS spectrum illustrates all elements present in the sample. Note that the signals of Cu and Si elements are derived from the copper microgrid for TEM test and the probe for EDS mapping, respectively.

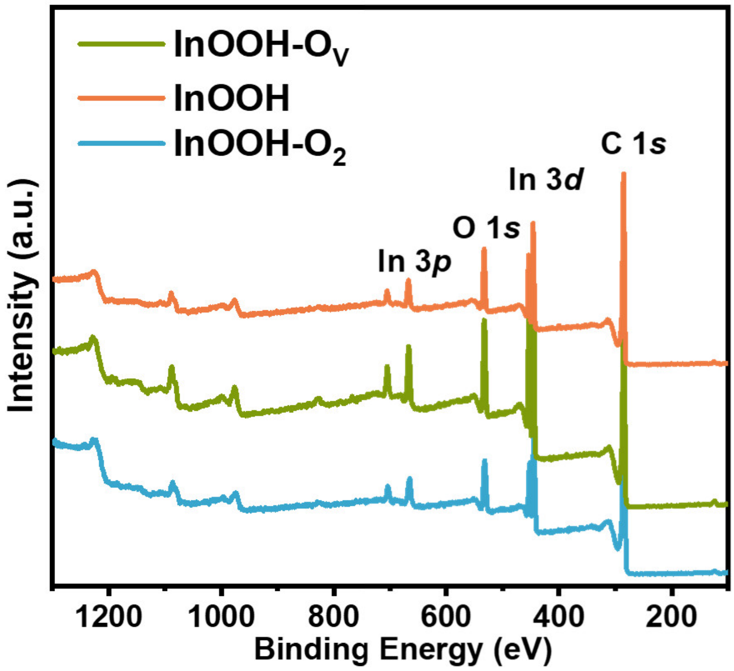

Supplementary Figure 5. XPS surveys. The three samples of InOOH, InOOH-O_V, and InOOH-O_2 presented similar signals of C, In, and O elements.

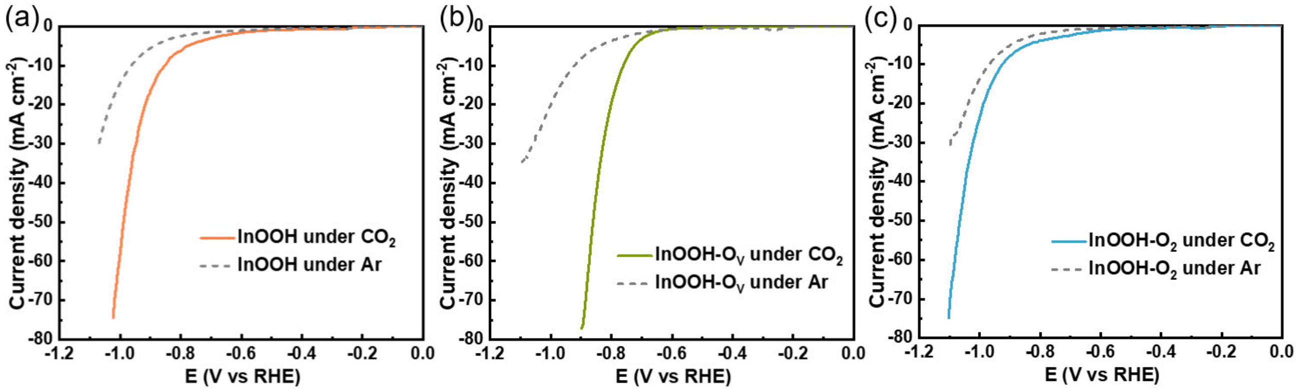

Supplementary Figure 6. The comparison of LSV curves in different atmosphere. The LSV curves of the prepared (a) InOOH, (b) InOOH-O_V, and (c) InOOH-O_2 under CO_2- and Ar- saturated electrolytes.

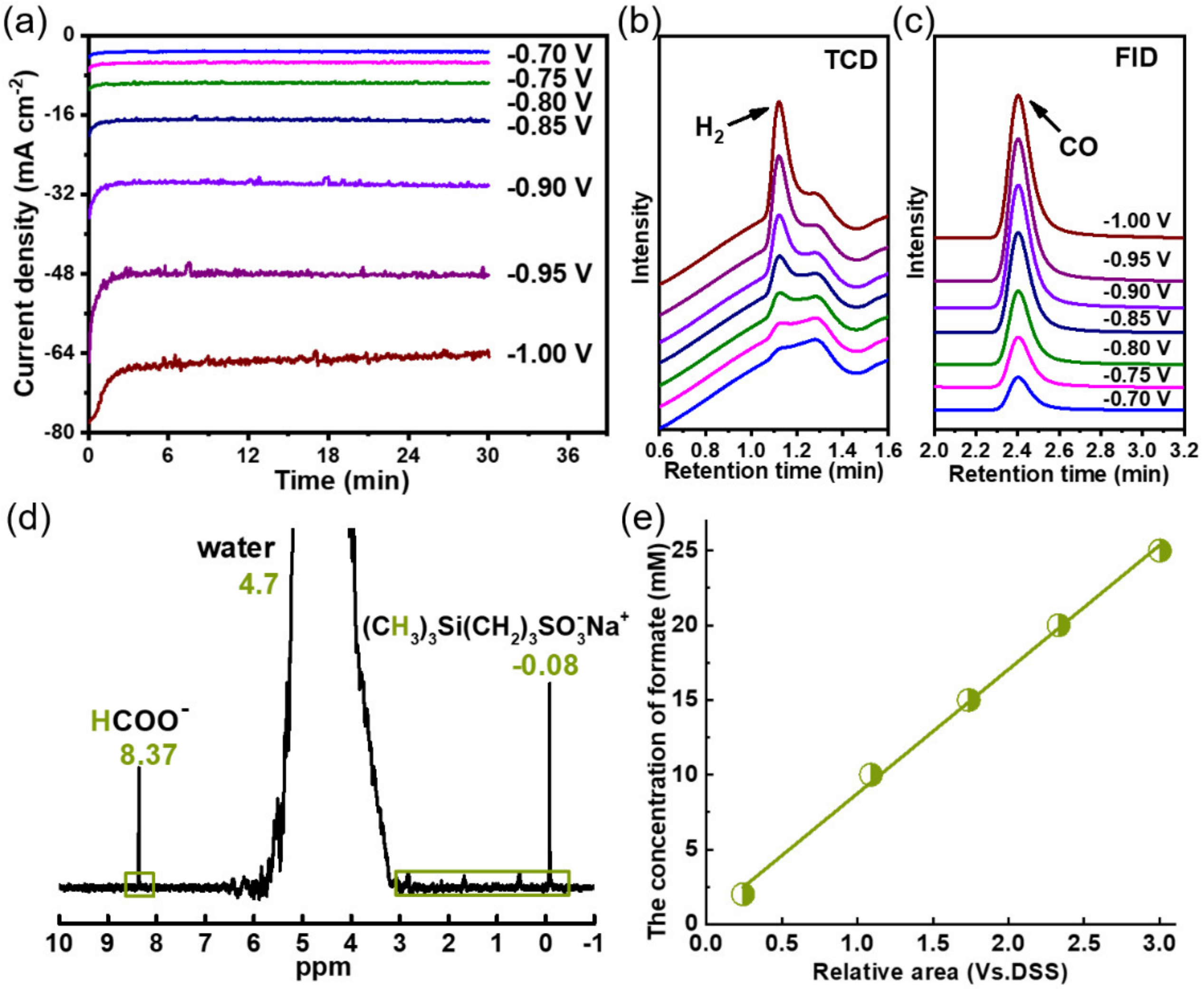

Supplementary Figure 7. The product analysis for CO_2RR. (a) The typical potentiostatic *i-t* curves over InOOH-O_V for CO_2RR. (b-c) The profiles of online GC tests showing H_2 and CO signals. (d) ^{1}H-NMR spectra for formate determination; (e) Linear relationship between the formate concentration and relative area *vs.* DSS.

To prepare the liquid sample for ^{1}H-NMR test, 0.5 mL electrolyte after electrolysis was extracted out to add in a solution containing 0.1 mL D_2O and 0.1 mL 6 mM DSS (1-Propanesulfonic acid 3-(trimethylsilyl) sodium salt). The calibration curve was achieved by testing sodium formate ($HCOONa{\cdot}2H_2O$) solution in different concentration from 2 mM to 25 mM. Then the formate concentration over relative area ratio of the formate peak to the DSS peak was plotted.

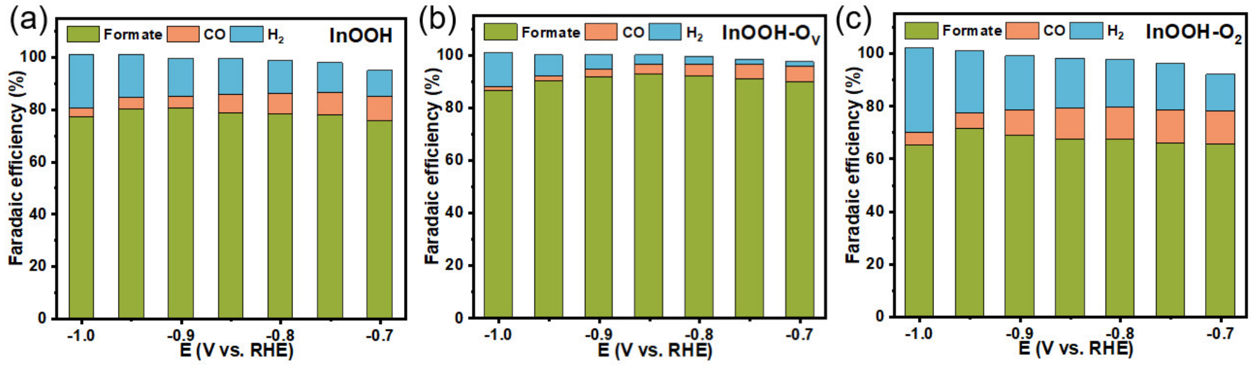

Supplementary Figure 8. The product distribution for CO_2RR. The FEs of formate, CO, and H_2 for CO_2RR tests under different potentials over three samples of (a) InOOH, (b) InOOH-O_V, and (c) InOOH-O_2.

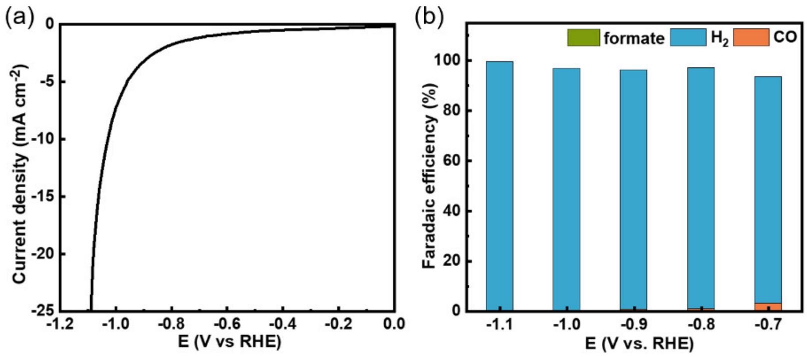

Supplementary Figure 9. The CO_2RR performance of Ar-plasma-treated CB. (a) LSV curve and (b) FEs of CO_2 reduction products on electrode with Ar-plasma-treated CB.

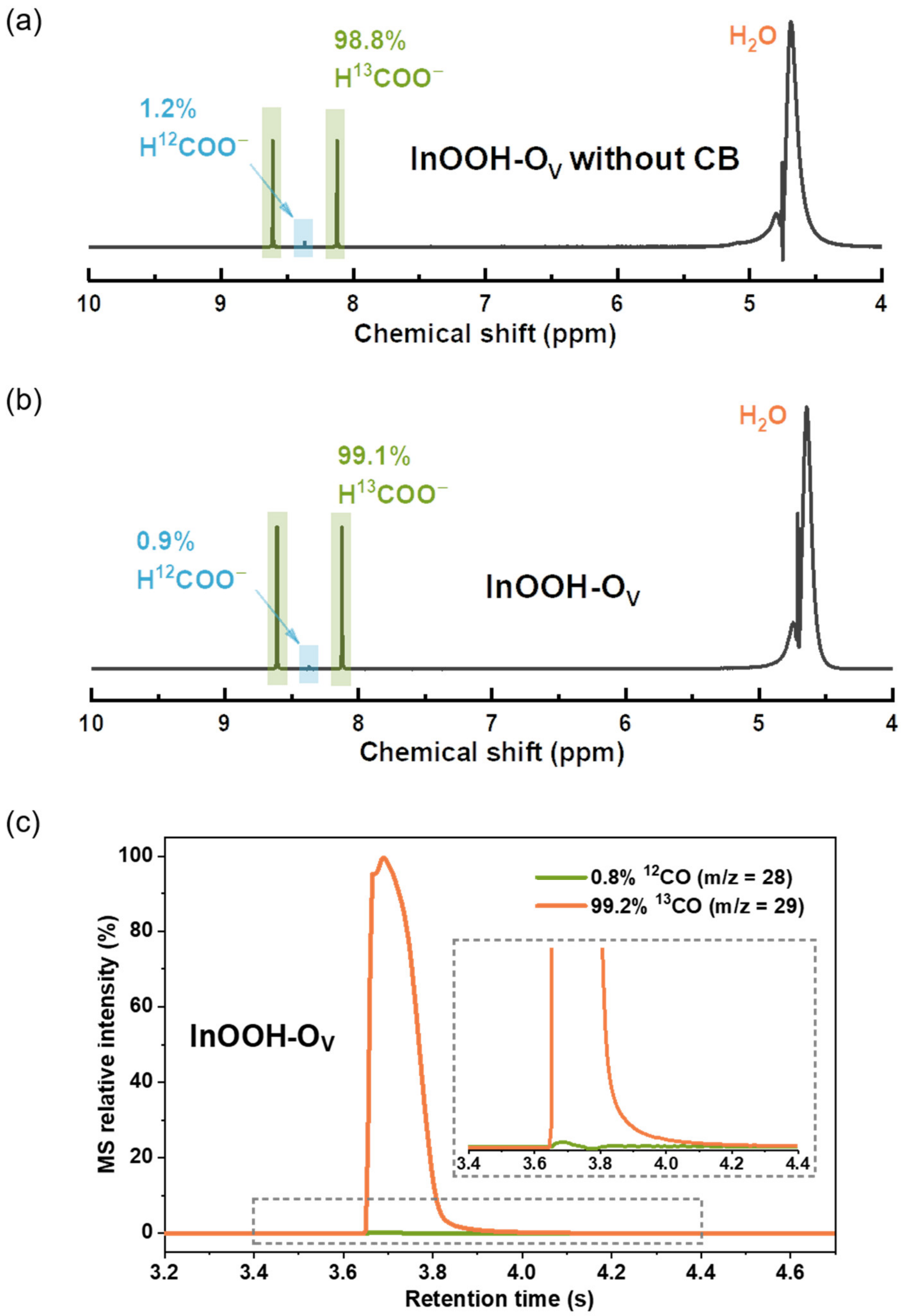

Supplementary Figure 10. The $^{13}CO_2$ isotope labling experiments. ^{1}H-NMR spectrum of produced $H^{13}COO^-$ and $H^{12}COO^-$ on InOOH-O$_V$ electrodes (a) without CB and (b) with CB as support at -0.85 V using $^{13}CO_2$ as the feeding gas. The overwhelming superiority in proportion of doublet of $H^{13}COO^-$ over the single peak of $H^{12}COO^-$ confirms the source of the obtained formate is $^{13}CO_2$ gas,[1] and the similar results for InOOH-O$_V$ electrodes with/without CB further exclude the probability of potential carbon contamination from CB. Note that the proportion of $^{13}CO_2$ we used for the isotope labeling experiment is primarily 99%, this is the reason why the tiny part of ^{12}C can be still detected in the products. (c) MS spectra of the CO product generated by InOOH-O$_V$ electrode with CB at -0.85 V using $^{13}CO_2$ as the feeding gas, showing the majority proportion of ^{13}CO (99.2%) and a negligible amount of ^{12}CO (0.8%), confirming the CO product is derived from the CO_2 reduction.

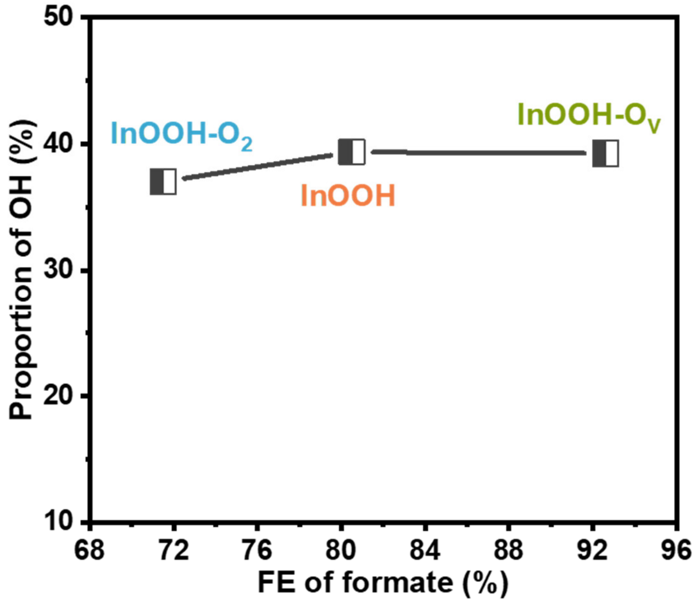

Supplementary Figure 11. The relationship of FE of formate and the propotion of OH species. No correlationship can be observed between the two factors.

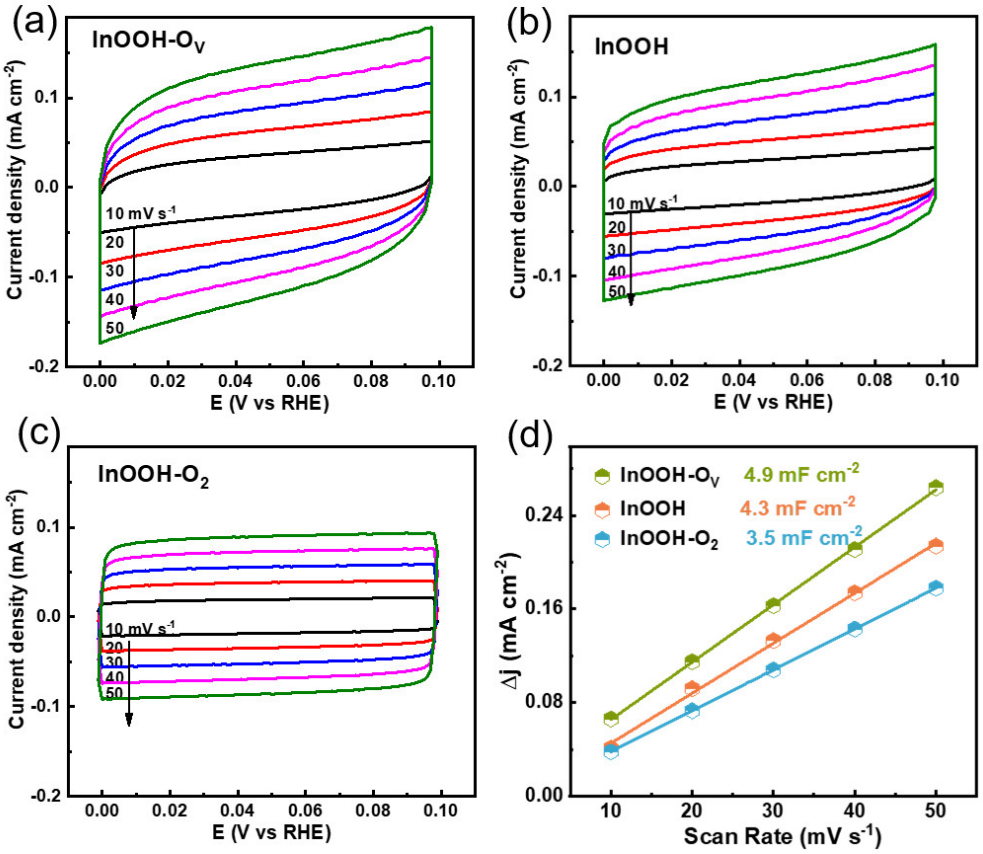

Supplementary Figure 12. ECSA measurements. (a-c) Cyclic voltammetry (CV) curves in the Non-faradaic regions for three samples of InOOH, InOOH-O_V, and InOOH-O_2. (d) The linear relation of Δj and scan rates for ECSA tests.

Electrochemical specific surface area (ECSA) was determined by CV method within the non-faradaic potential region between 0 and 0.1 V, as shown in Supplementary Figure 11. Specifically, ECSA was calculate from the formula of ECSA = $R_f S = (C_{dl}/60)S$,[2] where R_f denotes the roughness factor of the working electrode, S represents the geometric surface area of the gas diffusion electrode, and C_{dl} is the double-layer capacitance. For the samples in this work, the values of C_{dl} were obtained by plotting the differences of current density between the anode and cathode (Δj) at 0.05 V against the scan rates, equaling to the half of the linear slopes.

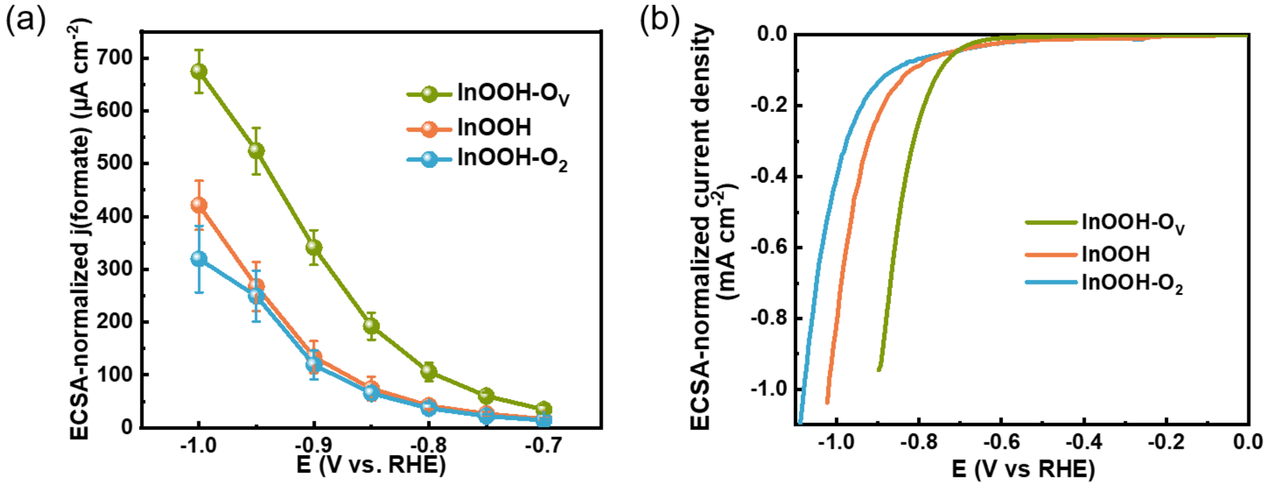

Supplementary Figure 13. Analysis of the effect of ECSA on CO_2RR performance. The ECSA normalized (a) $j_{formate}$ and (b) LSV curves. The error bars represent the standard deviations for three independent tests under the same conditions.

The total electrolysis current densities (i.e., LSV curves) and $j_{formate}$ of the three samples were normalized based on the ECSA from Supplementary Figure 12.

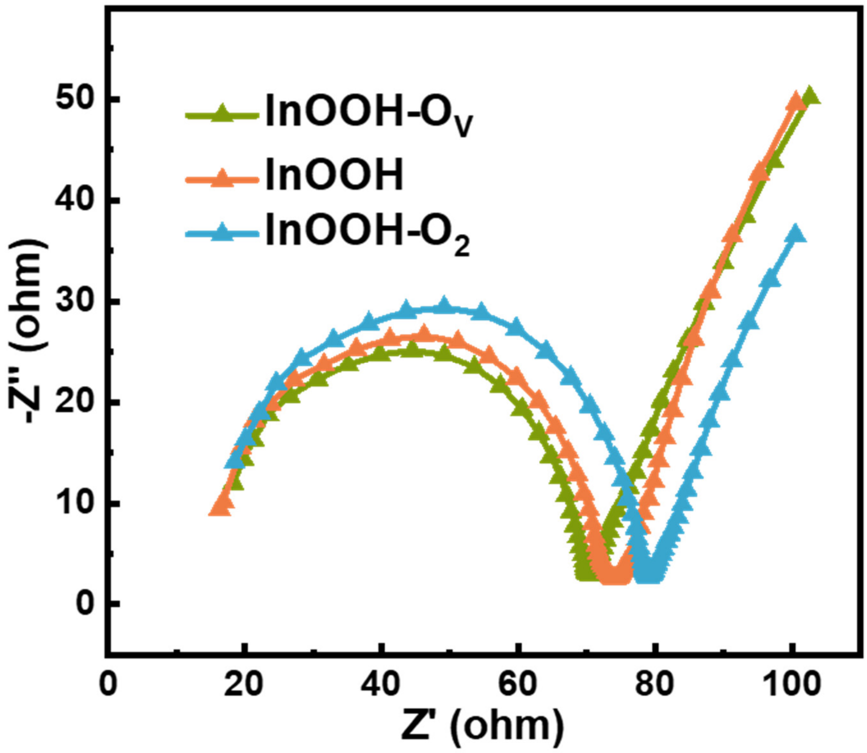

Supplementary Figure 14. EIS tests. The charge transfer impedance decreased from InOOH-O_2 through InOOH to InOOH-O_V.

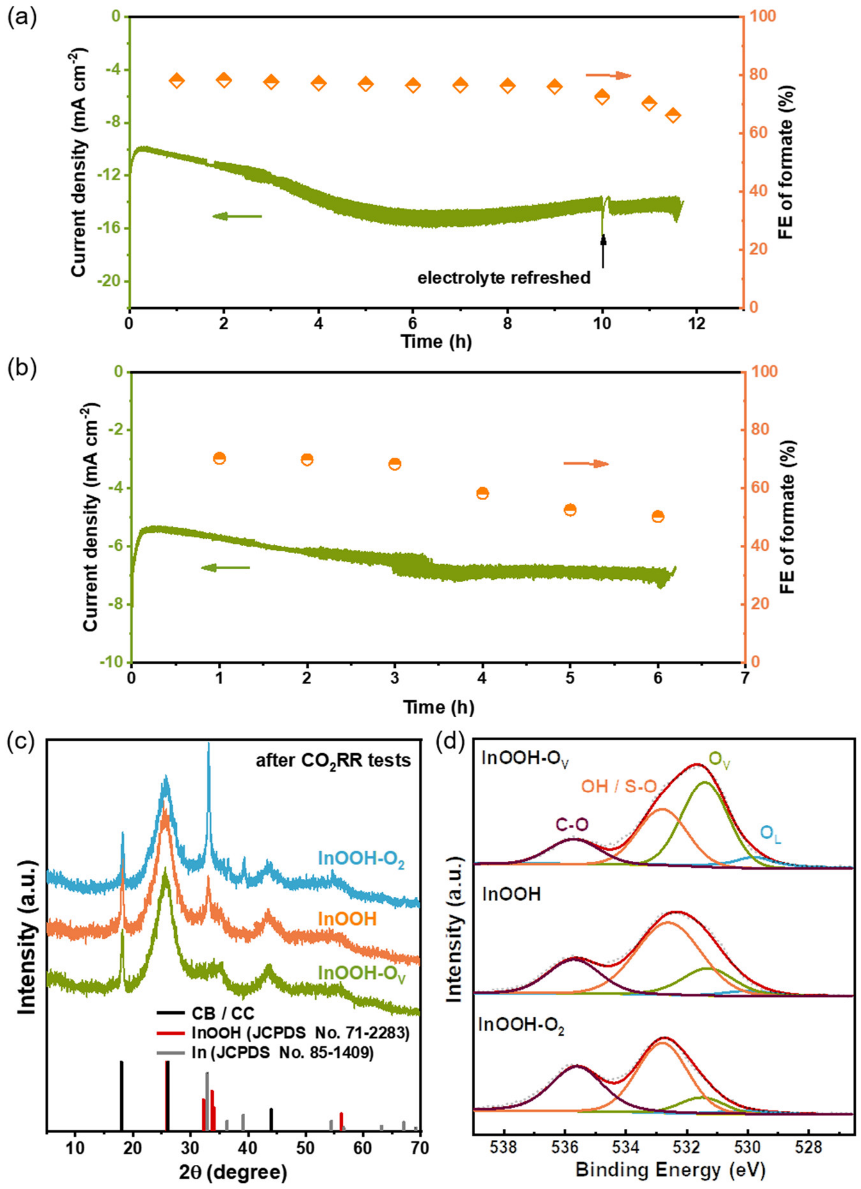

Supplementary Figure 15. The stability tests and physical charactrization of the samples before and after test for CO_2RR. The stability tests of (a) InOOH and (b) InOOH-O_2 for CO_2RR at -0.85 V. (c) XRD patterns and (d) O 1s XPS spectra of three electrodes after CO_2RR tests.

Note: The XRD patterns of electrodes after test show the signal of carbon cloth (CC). The deconvoled O 1s peak at binding energy of 532.8 eV represents an overlap of -OH and the sulfonic acid groups (labeled as S-O) in Nafion ionomer, and peak at binding energy of 535.7 eV is attributable to ether oxygen in Nafion ionomer (labeled as C-O).[3]

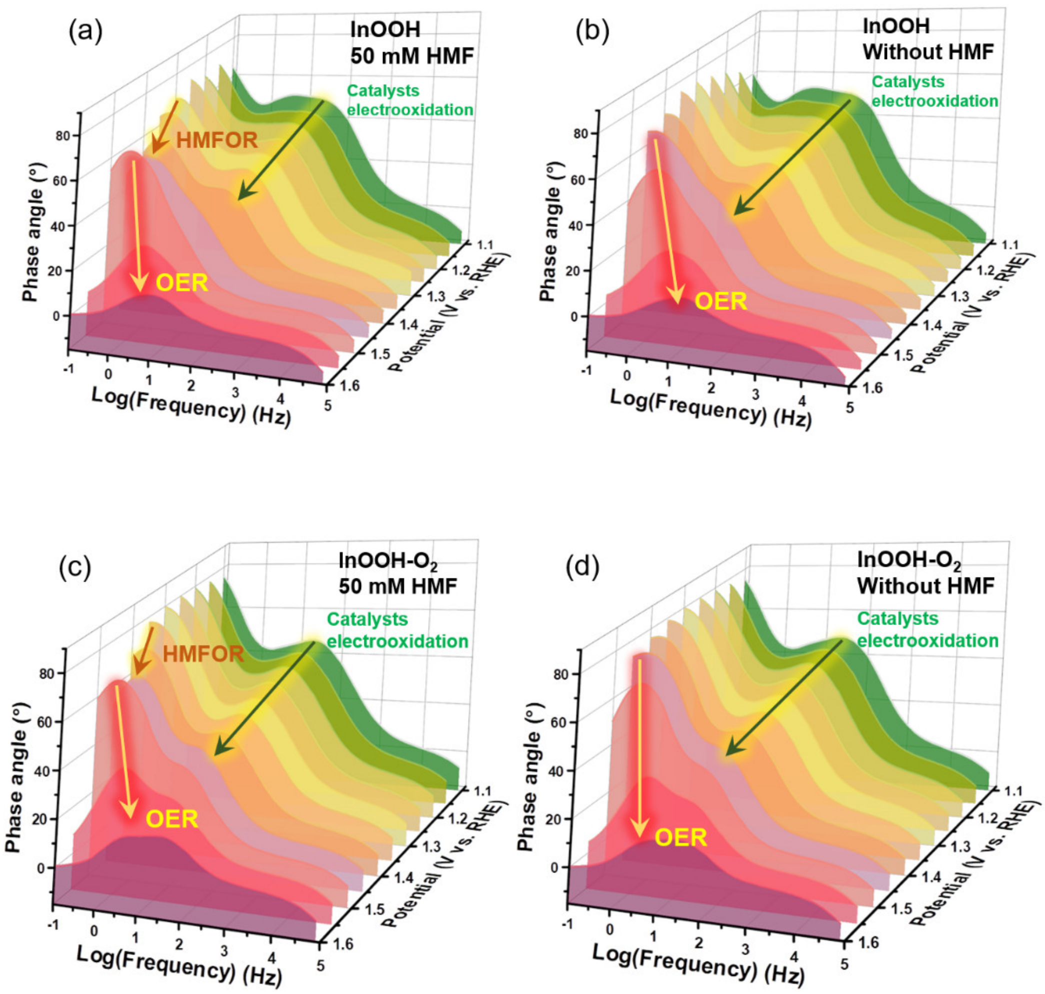

Supplementary Figure 16. The in situ EIS tests. Bode phase plots for EIS tests of (a-b) InOOH and (c-d) InOOH-O_2. The disappeared peaks for HMFOR were both observed for InOOH and InOOH-O_2 without adding 10 mM HMF. However, their HMFOR peaks were not as distinct as those of InOOH-O_V in Figure 4b of manuscript, depicting the weakened HMFOR activities due to the low content of O_V.

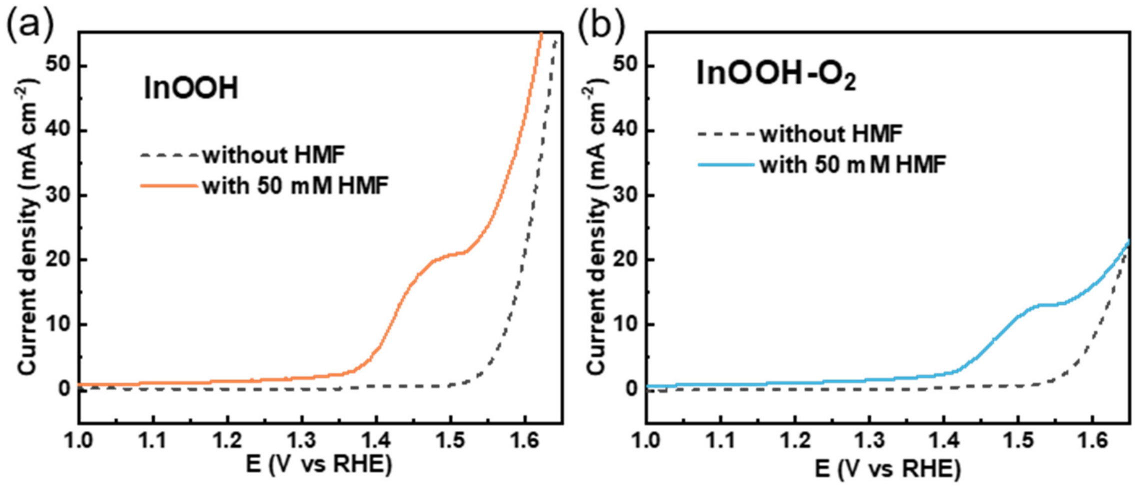

Supplementary Figure 17. The comparison of LSV curves with and without HMF. (a) InOOH, (b) InOOH-O_2 catalysts.

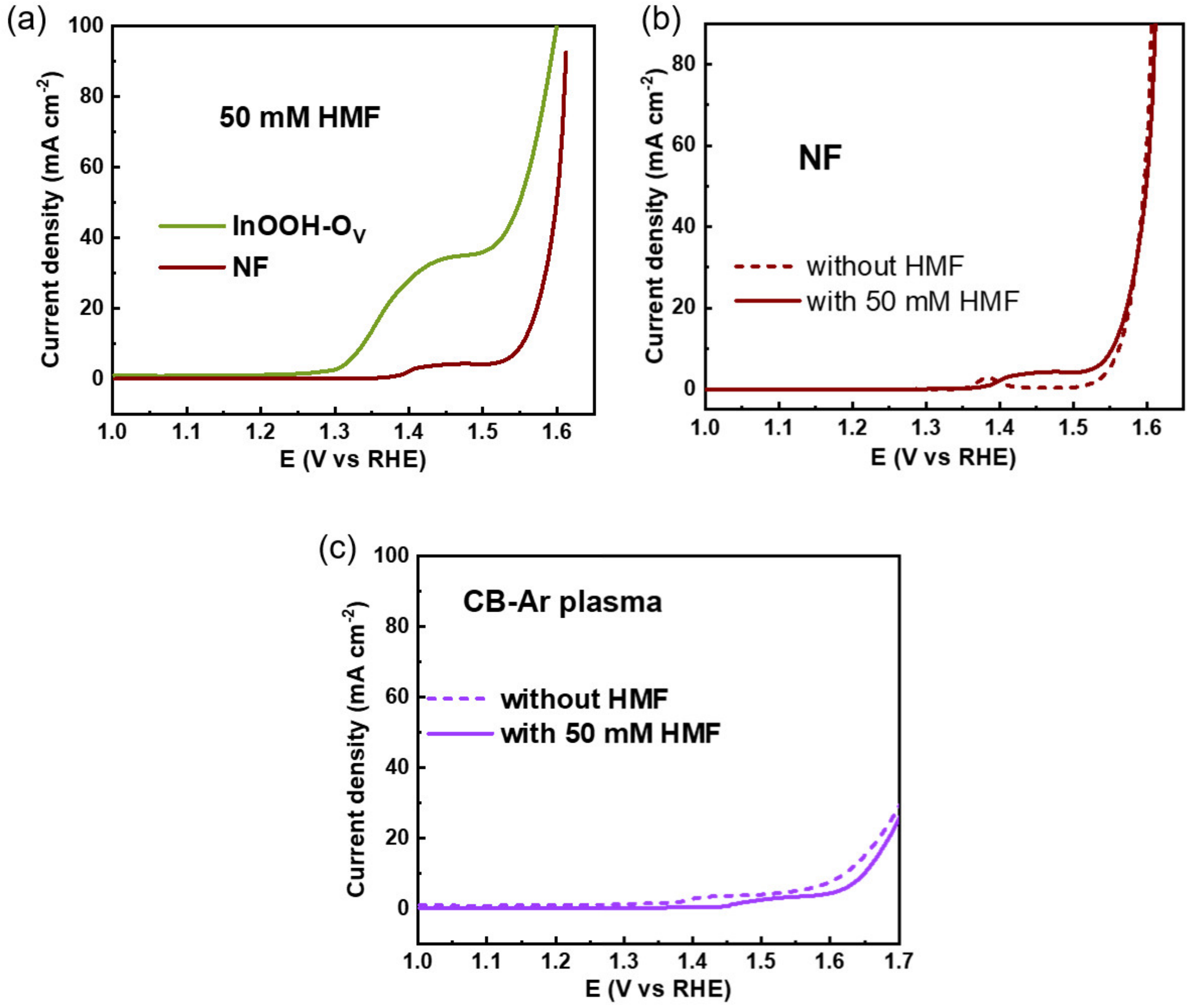

Supplementary Figure 18. The confirmation of HMFOR perofrmance for InOOH-O_V. (a) The comparison of LSV curves for HFMOR between InOOH-O_V and NF. LSV curves for (b) bare NF and (c) Ar-plasma treated CB on NF substrate with and without the addition of HMF.

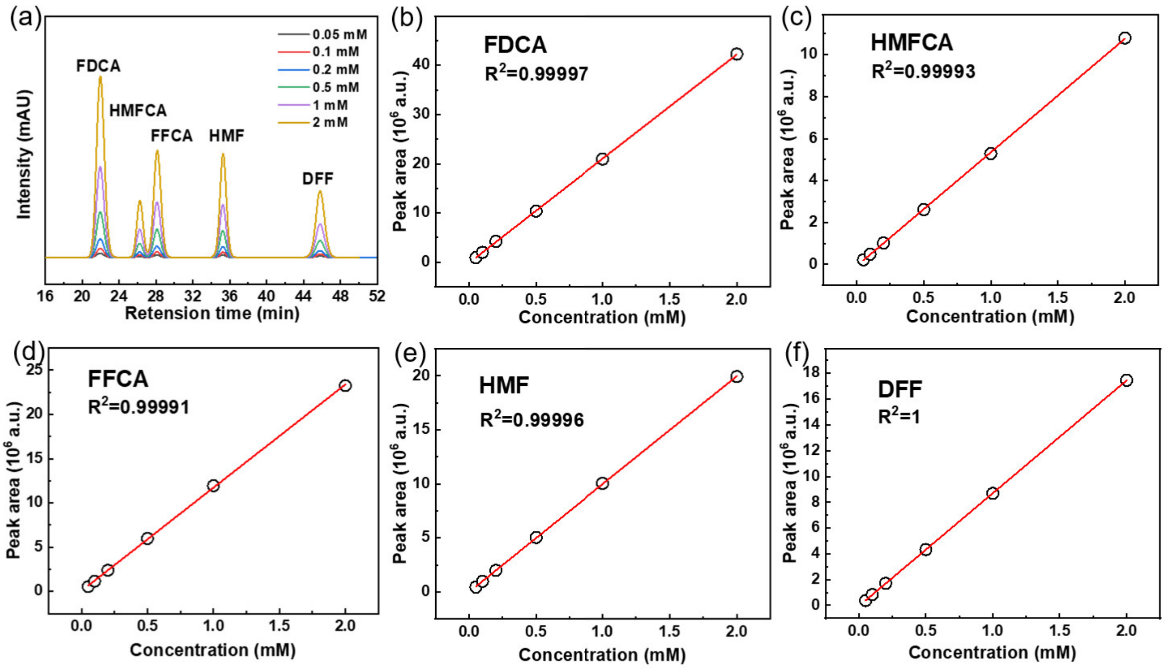

Supplementary Figure 19. The calibration curves of HMFOR products for HPLC tests. (a) The retention time curves. The linear fitting curves for (b) FDCA, (c) HMFCA, (d) FFCA, (e) HMF and (f) DFF.

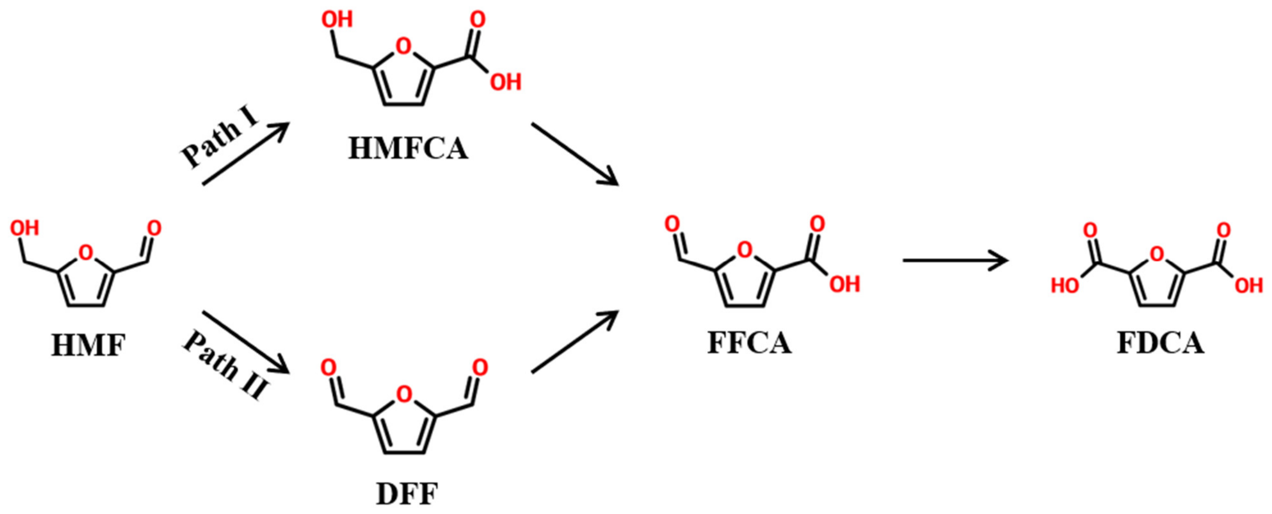

Supplementary Figure 20. Reaction pathways illustration for HMFOR processes. The two reaction pathways involves asynchronous oxidation steps of hydroxyl and aldehyde groups oxidation.

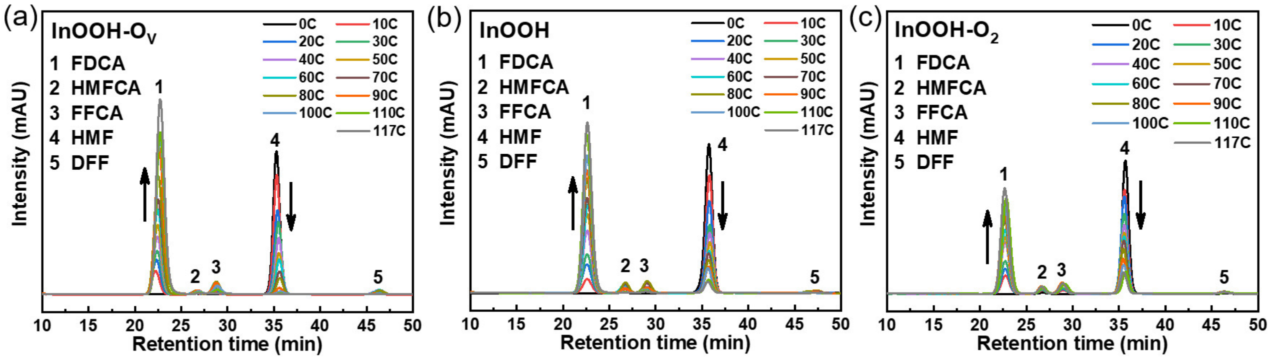

Supplementary Figure 21. The retention time curves for HPLC tests. (a) InOOH-O_V (b) InOOH, and (c) InOOH-O_2.

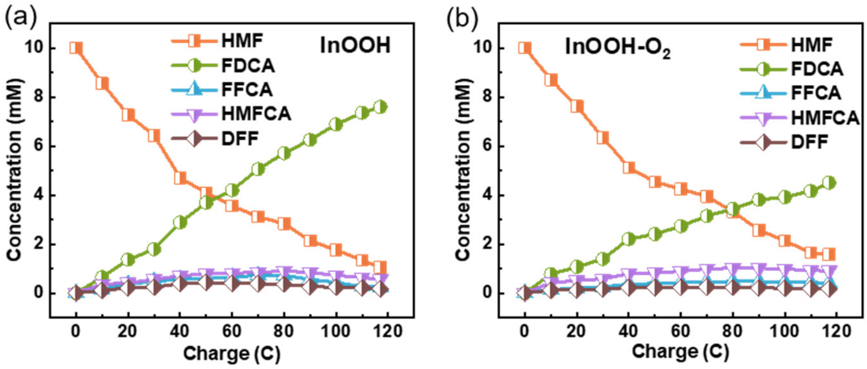

Supplementary Figure 22. The concentration variation of HMFOR products. (a) InOOH and (b) InOOH-O_2 catalysts.

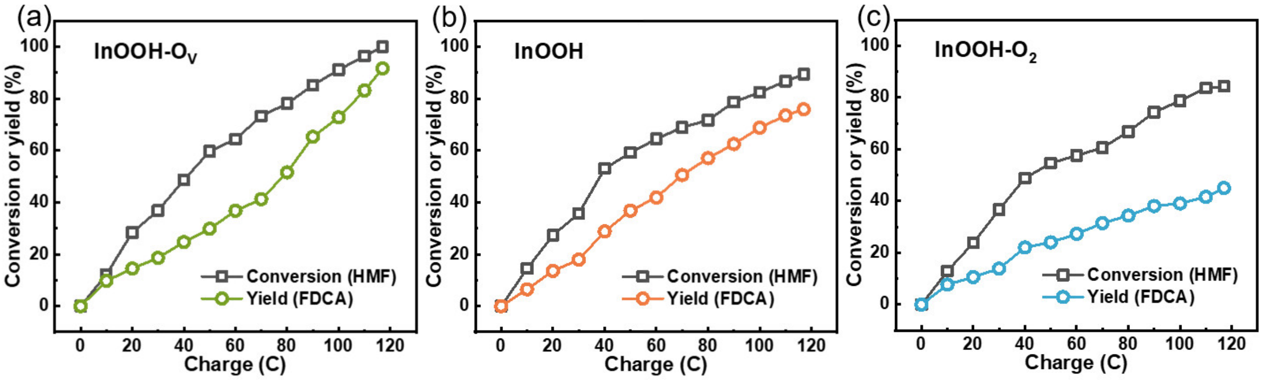

Supplementary Figure 23. The variation of HMF conversion and FDCA yield with the eletrolysis charge accumulation to 117 C. (a) InOOH-O_V, (b) InOOH , and (c) InOOH-O_2.

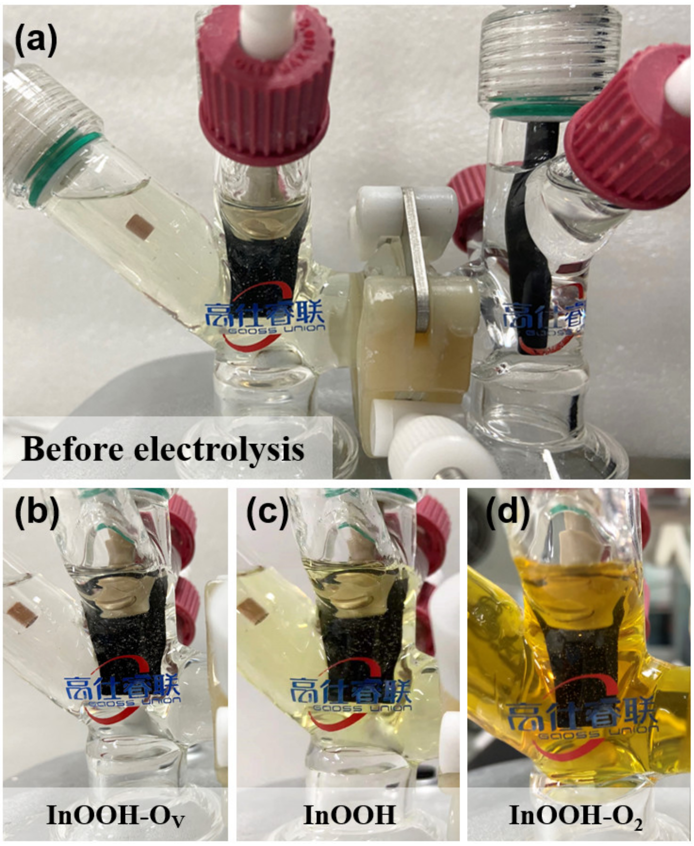

Supplementary Figure 24. The changing colors of anodic electrolyte before and after HMFOR tests for different samples of InOOH, InOOH-O$_V$, and InOOH-O$_2$. (a) The freshly prepared HMF solution (10 mM) appears the color of light-yellow. (b) HMF was oxidized efficiently and timely over InOOH-O$_V$, resulting in colorless electrolyte after electrolysis. (c,d) The sequentially weakened catalytic activites of HMFOR over InOOH and InOOH-O$_2$ lead to deeper and deeper electrolyte colors with electrolysis, as the strongly basic environment can cause HMF degradation through furan ring opening up and cross-polymerization to form brown, undesirable humins when HMF can not be consumed in time.[4]

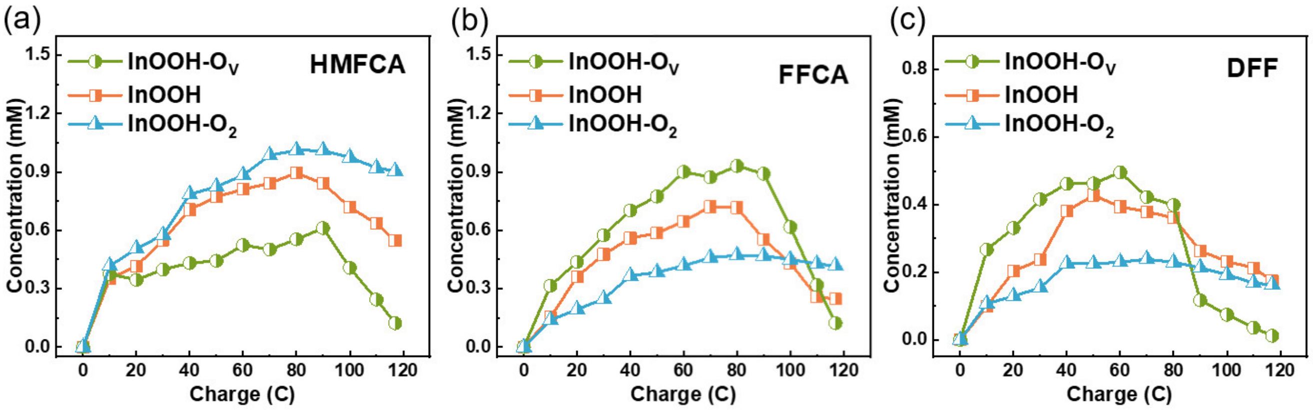

Supplementary Figure 25. Comparision of the concentration variations among samples of InOOH, InOOH-O_V, and InOOH-O_2. (a) HMFCA, (b) FFCA, and (c) DFF.

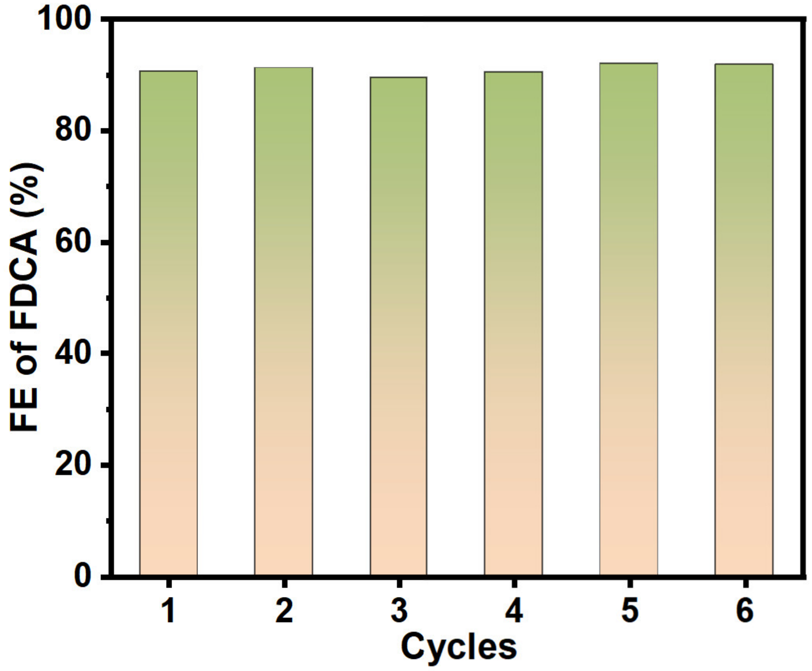

Supplementary Figure 26. Stability test. The FE of FDCA for six sequential electrolysis batches of HMFOR on InOOH-O_V at 1.48 V.

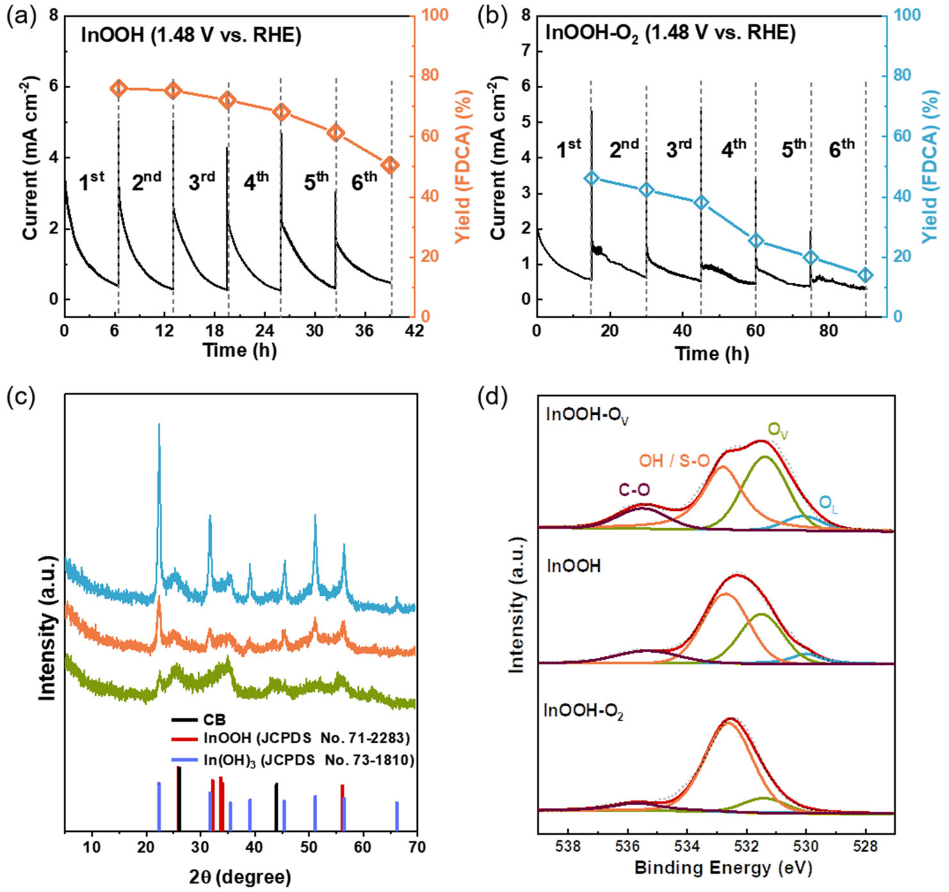

Supplementary Figure 27. The stability tests and physical charactrization of the samples before and after test for HMFOR. The stability tests of (a) InOOH and (b) InOOH-O_2 for HMFOR at 1.48 V. (c) The XRD patterns and (d) O 1s XPS spectra of three electrodes after HMFOR tests.

Note: The samples were removed from NF substrate by ultrasonic treatment and re-collected for characterization. The deconvoled O 1s peak at binding energy of 532.8 eV represents an overlap of -OH and the sulfonic acid groups (labeled as S-O) in Nafion ionomer, and peak at binding energy of 535.7 eV attributes to ether oxygen in Nafion ionomer (labeled as C-O).[3]

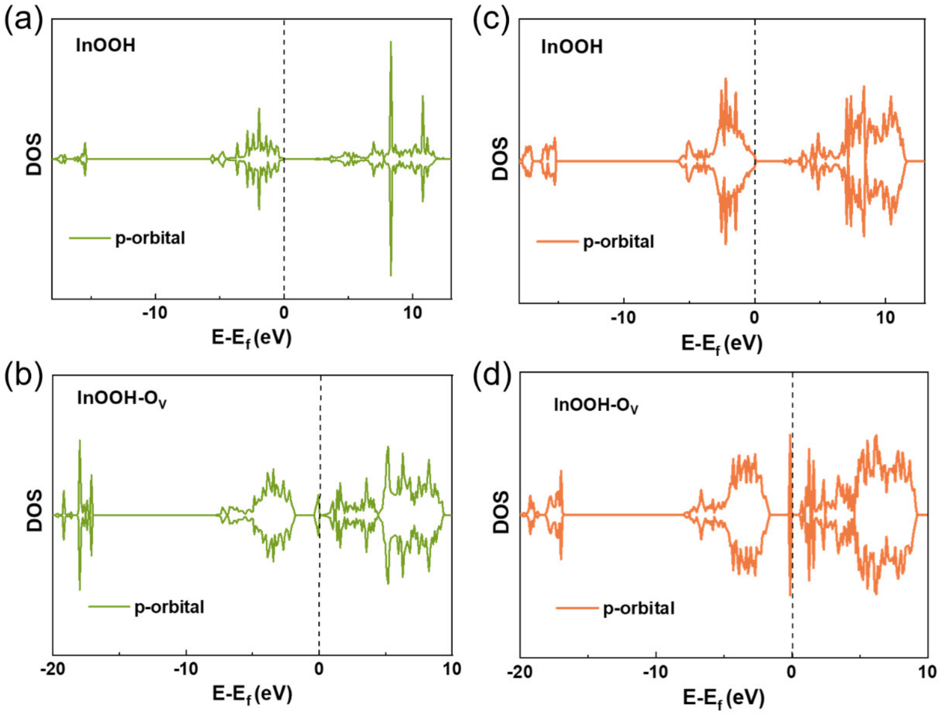

Supplementary Figure 28. The PDOS of the p-orbital on In atoms. The PBE-determined partial DOS of the p-orbital for In atom (a) on intact InOOH (110) plane and (b) adjcent to O_V site on InOOH-O_V. The HSE06-determined partial DOS of the p-orbital for In atom (c) on intact InOOH (110) plane and (d) adjcent to O_V site on InOOH-O_V.

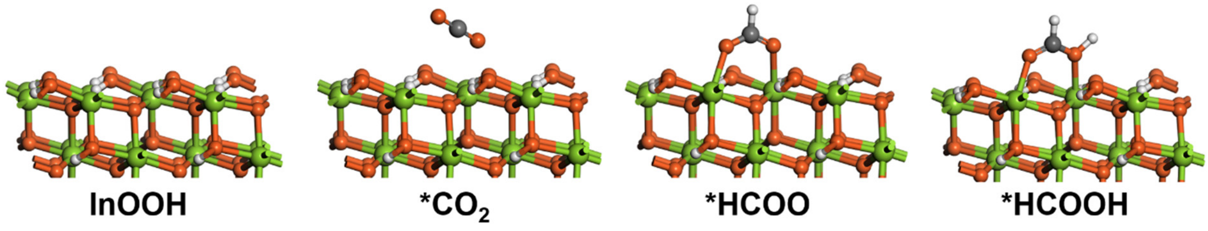

Supplementary Figure 29. Configurations of intermediates. The simulated results on models of InOOH (110) during CO_2RR.

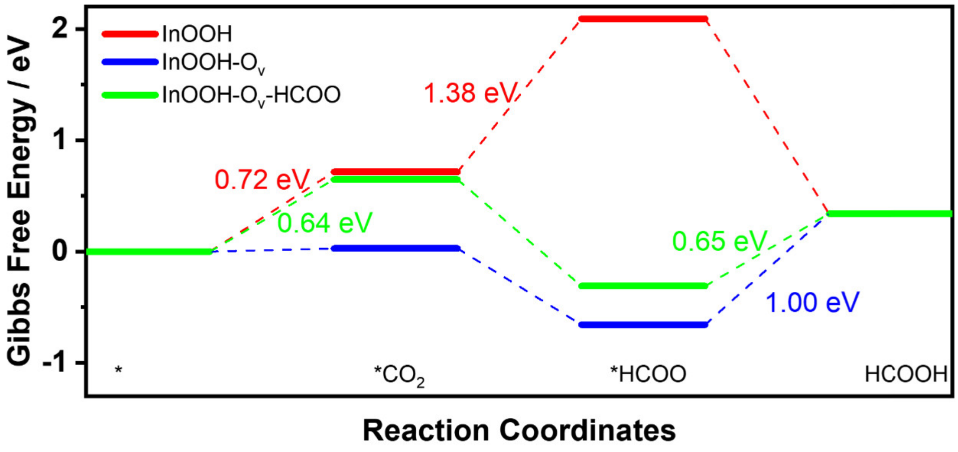

Supplementary Figure 30. Free energy diagrams for CO_2RR. RPBE was used to explore the functional sensitivity.

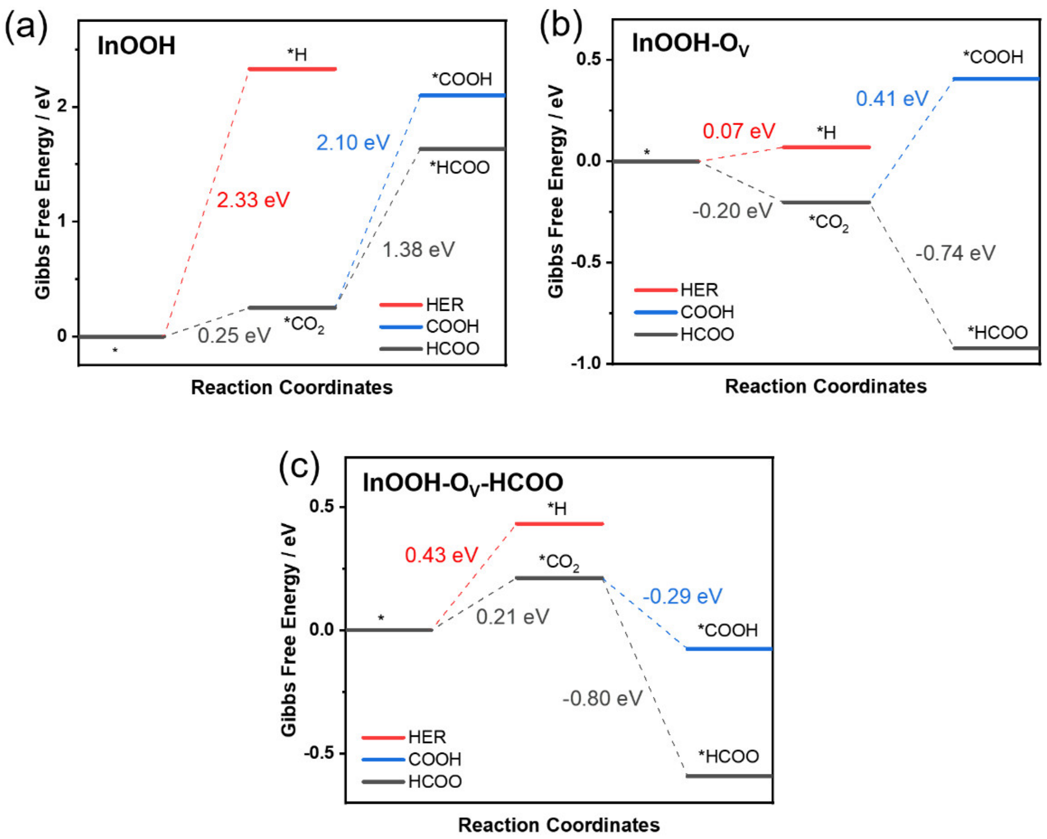

Supplementary Figure 31. Free energy diagrams for HER and CO_2RR. (a) InOOH, (b) InOOH-O_V, and (c) InOOH-O_V-HCOO.

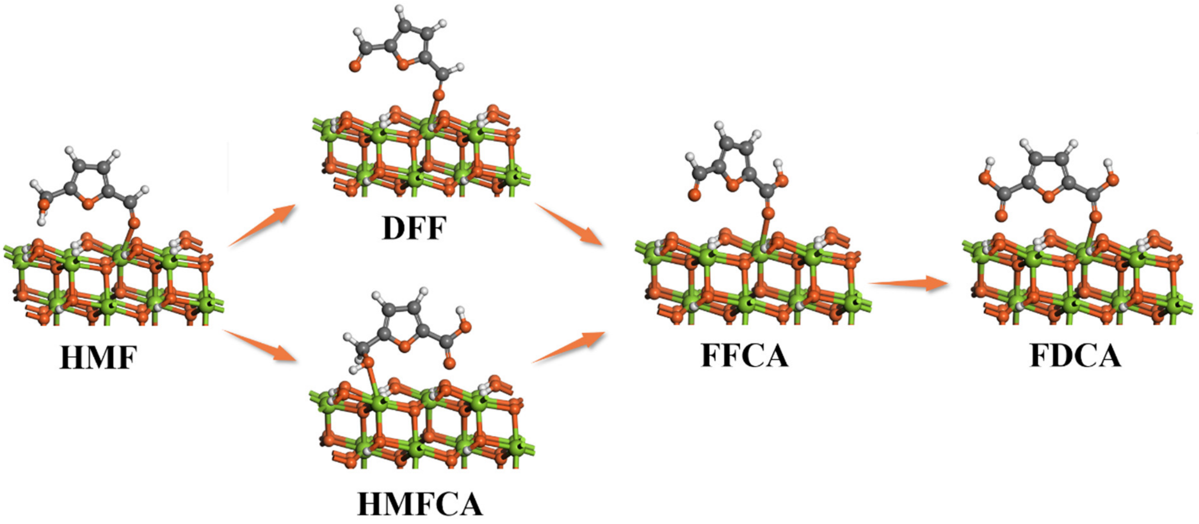

Supplementary Figure 32. Configurations of intermediates. The simulated results on models of InOOH (110) during HMFOR.

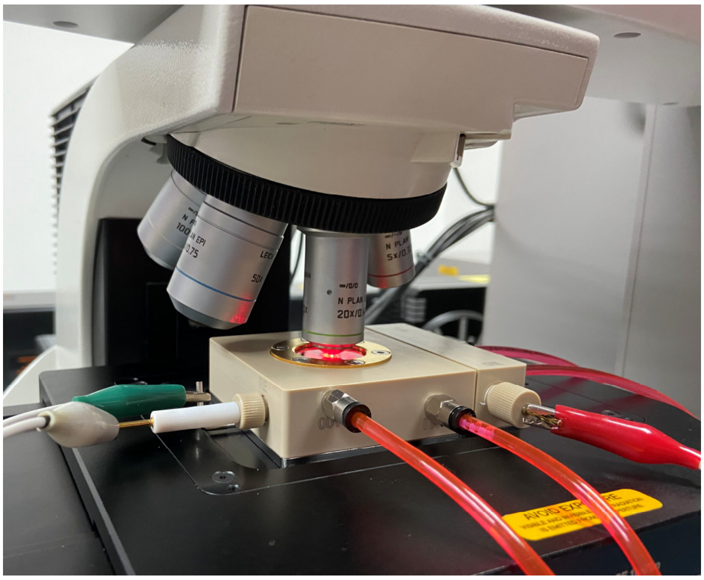

Supplementary Figure 33. Operando Raman spectra acquisition. The H-shape electrolysis cell with an optical quartz window was used.

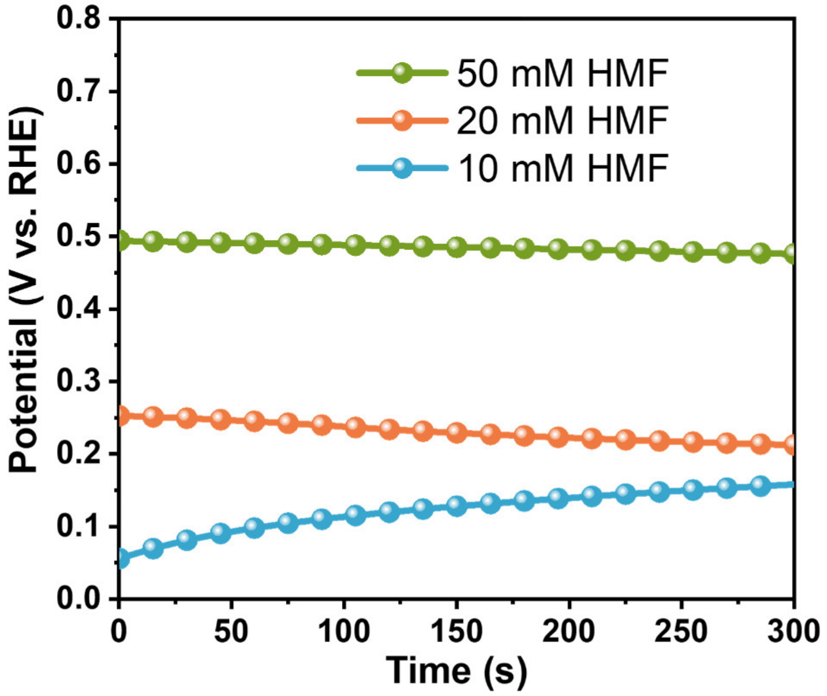

Supplementary Figure 34. The OCP measurements for InOOH-O$_V$. Different HMF concentrations of 10, 20, and 50 mM in 1 M KOH were used under in situ Raman spectroscopy acquisition condition. The bigger OCP for the higher HMF concentration indicated the more occupied O$_V$ sites by HMF molecules.

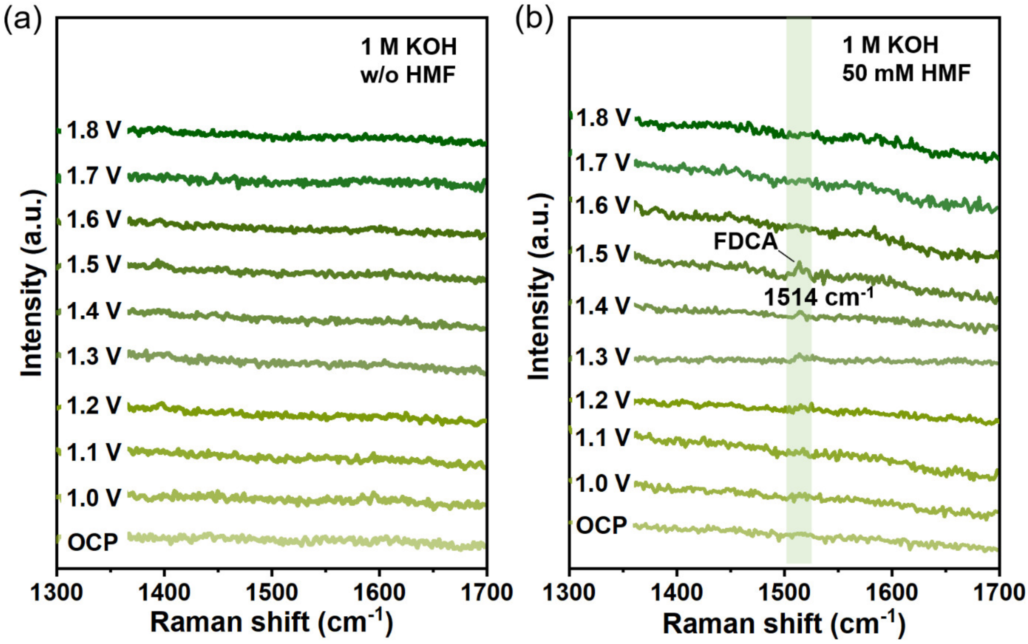

Supplementary Figure 35. The in situ Raman spectroscopies. The potential-dependent in situ Raman spectroscopies obtained in the range of 1300 and 1700 cm^{-1} over InOOH-O_V in (a) 1 M KOH without adding HMF and (b) with 50 mM HMF.

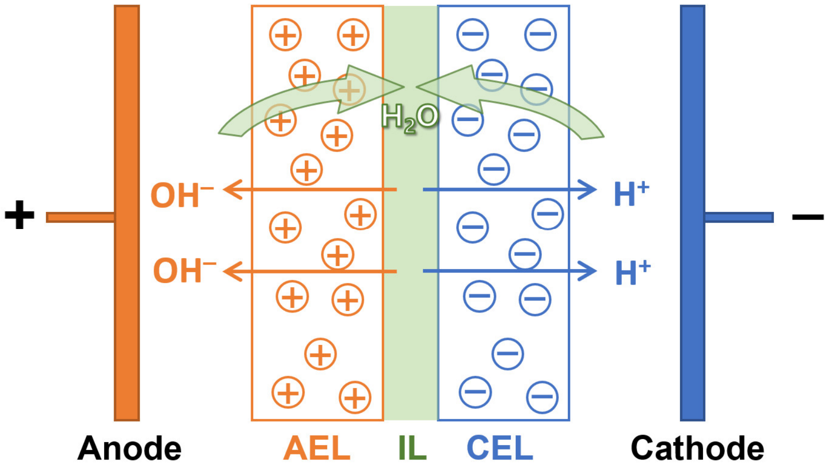

Supplementary Figure 36. The illustration of BPM structure. A typical BPM consists of laminated films of anion-exchange layer (AEL) and cation-exchange layer (CEL) with a bipolar interfacial layer (IL) formed between that allows selective diffusion of protons and hydroxide anions towards the negative and positive electrode, respectively.[5]

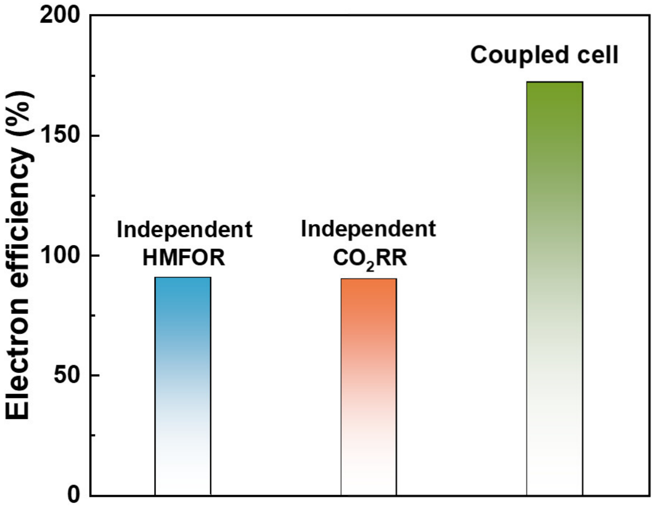

Supplementary Figure 37. The electron efficiency comparison of independent HMFOR, CO_2RR, and coupled cell. "Combined electron efficiency" represents the overall utilization efficiency of electrons that are transferred between cathode and anode to produce target value-added chemicals (formate and FDCA) in terms of the whole electrolysis system. The higher the "combined electron efficiency", the higher the utilization of electricity.

Table S1. The atomic contents of different elements in the three samples

Sample	C (at. %)	O (at. %)	In (at. %)	Mass fraction of InOOH
InOOH	84.8	12.0	3.3	32.1 %
InOOH-O_V	86.1	10.7	3.3	31.9 %
InOOH-O_2	82.7	13.7	3.6	34.8 %

Table S2. The area fraction of deconvolved O 1s peaks of InOOH, InOOH-O_V, and InOOH-O_2

Sample	O_L	O_V	OH
InOOH	28.0%	32.6%	39.4%
InOOH-O_V	19.9%	40.9%	39.3%
InOOH-O_2	34.0%	29.0%	37.0%

Table S3. Recent works using metal oxides as catalysts for CO_2RR to formate testing in H-shape cell at ambient condition

Catalysts	Electrolyte	Potential	FE_{max} (%)	j(formate) (mA cm^{-2})	Ref.
CoO_x	0.1 M Na_2SO_4	-0.25 V vs.RHE	90	9	6
SW-Cu_2O/Cu	0.5 M $KHCO_3$	-0.84 V vs RHE	27.1	-	7
PVP@Cu_2O	0.1 M K_2SO_4	-0.65 V vs RHE	77.6	-	8
In_2O_3-rGO	0.1 M $KHCO_3$	-1.20 V vs.RHE	84.6	22	9
SnO_2/Graphene	0.1 M $NaHCO_3$	-1.8 V vs.SCE	93.6	10.2	10
SnO_2 QWs	0.1 M $KHCO_3$	-1.16 V vs.RHE	87.3	13.7	11
SnO_2 nanosheets	0.5 M $NaHCO_3$	-1.6 V vs.SCE	87	45	12
V_o-SnO_2	0.5 M $NaHCO_3$	-0.7 V vs.RHE	92.4	~2.5	13
Co_3O_4 layers	0.1 M $KHCO_3$	-0.87 V vs.SCE	87.6	2.7	2
InO_x nanoribbons	0.5 M $NaHCO_3$	-0.7 V vs.RHE	91.7	5	14
SnO_2 nanowires	0.1 M $KHCO_3$	-0.8 V vs.RHE	80.1	4.8	15
V_O-rich N-SnO_2	0.1 M $KHCO_3$	-0.9 V vs.RHE	83	6.7	16
**InOOH-O_V**	0.1 M $KHCO_3$	-0.85 V vs.RHE	**92.6**	**16.0**	**This work**
**InOOH-O_V**	0.1 M $KHCO_3$	-0.95 V vs.RHE	**90.0**	**43.7**	**This work**
**InOOH-O_V**	0.1 M $KHCO_3$	-1.00 V vs.RHE	**86.4**	**56.2**	**This work**

Table S4. Recent works on catalysts for HMFOR to FDCA

Catalysts	Onset potential (V *vs.* RHE)	HMF conversion (%)	FDCA yield (%)	Ref.
InOOH-O_V	**1.30**	**98.5**	**91.6**	**This work**
Ni_2P NPA/NF	1.35	100	98	17
Ni_3S_2/NF	1.35	100	98	18
hp-Ni	1.35	100	98	19
NiFe LDH	1.25	98	98	20
Ni_xB/NF	1.38	100	98.5	21
$NiCo_2O_4$/NF	1.35	100	90	22
$CuNi(OH)_2$/C	1.38	98.8	93.3	23
NiOOH/FTO	NA	99.8	96	24
CoOOH/FTO	NA	95.5	35.1	24
FeOOH/FTO	NA	16	1.59	24
VN/NiF	1.36	98	96	25
NiSe@NiO_x	1.35	100	99	26
MoO_2-FeP@C	~1.35	99.4	98	27
Ni_3N@C	1.35	100	98	28
NiCoFe-LDHs	~1.50	~84.9	~90	29
CoO-$CoSe_2$	1.30	100	99	30
Vo-Co3O4	~1.30	NA	91.9	31
P-HEOs	~1.30	99	97.4	32

References

1. Cheng H, *et al.* Surface Nitrogen-Injection Engineering for High Formation Rate of CO(2) Reduction to Formate. *Nano Lett.* **20**, 6097-6103 (2020).
2. Gao S, *et al.* Atomic layer confined vacancies for atomic-level insights into carbon dioxide electroreduction. *Nat. Commun.* **8**, 14503 (2017).
3. Vijayakumar M, *et al.* Spectroscopic investigations of the fouling process on Nafion membranes in vanadium redox flow batteries. *J. Membr. Sci.* **366**, 325-334 (2011).
4. Giannakoudakis DA, Colmenares JC, Tsiplakides D, Triantafyllidis KS. Nanoengineered Electrodes for Biomass-Derived 5-Hydroxymethylfurfural Electrocatalytic Oxidation to 2,5-Furandicarboxylic Acid. *ACS Sustain. Chem. Eng.* **9**, 1970-1993 (2021).
5. Giesbrecht PK, Freund MS. Recent advances in bipolar membrane design and applications. *Chem. Mater.* **32**, 8060-8090 (2020).
6. Gao S, *et al.* Partially oxidized atomic cobalt layers for carbon dioxide electroreduction to liquid fuel. *Nature* **529**, 68-71 (2016).
7. Li J, *et al.* Electroreduction of CO(2) to Formate on a Copper-Based Electrocatalyst at High Pressures with High Energy Conversion Efficiency. *J. Am. Chem. Soc.* **142**, 7276-7282 (2020).
8. Tan D, Wulan B, Ma J, Cao X, Zhang J. Interface Molecular Functionalization of Cu_2O for Synchronous Electrocatalytic Generation of Formate. *Nano Lett.* **22**, 6298-6305 (2022).
9. Zhang Z, *et al.* Enhanced Electrocatalytic Reduction of CO_2 via Chemical Coupling between Indium Oxide and Reduced Graphene Oxide. *Nano Lett.* **19**, 4029-4034 (2019).
10. Zhang S, Kang P, Meyer TJ. Nanostructured tin catalysts for selective electrochemical reduction of carbon dioxide to formate. *J. Am. Chem. Soc.* **136**, 1734-1737 (2014).
11. Liu S, Xiao J, Lu XF, Wang J, Wang X, Lou XWD. Efficient Electrochemical Reduction of CO_2 to HCOOH over Sub-2 nm SnO_2 Quantum Wires with Exposed Grain Boundaries. *Angew. Chem. Int. Ed.* **58**, 8499-8503 (2019).
12. Li F, Chen L, Knowles GP, MacFarlane DR, Zhang J. Hierarchical Mesoporous SnO_2 Nanosheets on Carbon Cloth: A Robust and Flexible Electrocatalyst for CO_2 Reduction with High Efficiency and Selectivity. *Angew. Chem. Int. Ed.* **56**, 505-509 (2017).
13. Liu G, *et al.* Black reduced porous SnO_2 nanosheets for CO_2 electroreduction with high formate selectivity and low overpotential. *Appl. Catal. B-Environ.* **260**, 118134 (2020).
14. Zhang J, Yin R, Shao Q, Zhu T, Huang X. Oxygen Vacancies in Amorphous InOx Nanoribbons Enhance CO_2 Adsorption and Activation for CO_2 Electroreduction. *Angew. Chem. Int. Ed.* **58**, 5609-5613 (2019).
15. Kumar B, *et al.* Reduced SnO_2 Porous Nanowires with a High Density of Grain Boundaries as Catalysts for Efficient Electrochemical CO_2-into-HCOOH Conversion. *Angew. Chem. Int. Ed.* **56**, 3645-3649 (2017).
16. Li Z, *et al.* Elucidation of the Synergistic Effect of Dopants and Vacancies on Promoted Selectivity for CO_2 Electroreduction to Formate. *Adv. Mater.* **33**, e2005113 (2021).
17. You B, Jiang N, Liu X, Sun Y. Simultaneous H2 Generation and Biomass Upgrading in Water by an Efficient Noble-Metal-Free Bifunctional Electrocatalyst. *Angew. Chem. Int. Ed.* **55**, 9913-9917 (2016).
18. You B, Liu X, Jiang N, Sun Y. A General Strategy for Decoupled Hydrogen Production from Water Splitting by Integrating Oxidative Biomass Valorization. *J. Am. Chem. Soc.* **138**, 13639-13646 (2016).
19. You B, Liu X, Liu X, Sun Y. Efficient H_2 Evolution Coupled with Oxidative Refining of Alcohols via A Hierarchically Porous Nickel Bifunctional Electrocatalyst. *ACS Catal.* **7**, 4564-4570 (2017).
20. Liu W-J, Dang L, Xu Z, Yu H-Q, Jin S, Huber GW. Electrochemical Oxidation of 5-Hydroxymethylfurfural with NiFe Layered Double Hydroxide (LDH) Nanosheet Catalysts. *ACS Catal.* **8**, 5533-5541 (2018).
21. Barwe S, *et al.* Electrocatalytic Oxidation of 5-(Hydroxymethyl)furfural Using High-Surface-Area Nickel Boride. *Angew. Chem. Int. Ed.* **57**, 11460-11464 (2018).
22. Gao L, *et al.* Hierarchical Nickel-Cobalt-Based Transition Metal Oxide Catalysts for the Electrochemical Conversion of Biomass into Valuable Chemicals. *ChemSusChem* **11**, 2547-2553 (2018).
23. Chen H, *et al.* Cu−Ni Bimetallic Hydroxide Catalyst for Efficient Electrochemical Conversion of 5‐Hydroxymethylfurfural to 2,5‐Furandicarboxylic Acid. *ChemElectroChem* **6**, 5797-5801 (2019).

24. Taitt BJ, Nam D-H, Choi K-S. A Comparative Study of Nickel, Cobalt, and Iron Oxyhydroxide Anodes for the Electrochemical Oxidation of 5-Hydroxymethylfurfural to 2,5-Furandicarboxylic Acid. *ACS Catal.* **9**, 660-670 (2018).
25. Li S*, et al.* Biomass Valorization via Paired Electrosynthesis Over Vanadium Nitride‐Based Electrocatalysts. *Adv. Funct. Mater.* **29**, 1904780 (2019).
26. Gao L*, et al.* NiSe@NiOx core-shell nanowires as a non-precious electrocatalyst for upgrading 5-hydroxymethylfurfural into 2,5-furandicarboxylic acid. *Appl. Catal. B-Environ.* **261**, 118235 (2020).
27. Yang G*, et al.* Interfacial Engineering of MoO2 -FeP Heterojunction for Highly Efficient Hydrogen Evolution Coupled with Biomass Electrooxidation. *Adv. Mater.* **32**, e2000455 (2020).
28. Zhang N*, et al.* Electrochemical Oxidation of 5-Hydroxymethylfurfural on Nickel Nitride/Carbon Nanosheets: Reaction Pathway Determined by In Situ Sum Frequency Generation Vibrational Spectroscopy. *Angew. Chem. Int. Ed.* **58**, 15895-15903 (2019).
29. Zhang M, Liu Y, Liu B, Chen Z, Xu H, Yan K. Trimetallic NiCoFe-Layered Double Hydroxides Nanosheets Efficient for Oxygen Evolution and Highly Selective Oxidation of Biomass-Derived 5-Hydroxymethylfurfural. *ACS Catal.* **10**, 5179-5189 (2020).
30. Huang X*, et al.* Enhancing the Electrocatalytic Activity of CoO for the Oxidation of 5-hydroxymethylfurfural by Introducing Oxygen Vacancies. *Green Chem.* **22**, 843-849 (2020).
31. Lu Y*, et al.* Tailoring Competitive Adsorption Sites by Oxygen-Vacancy on Cobalt Oxides to Enhance the Electrooxidation of Biomass. *Adv. Mater.* **34**, 2107185 (2021).
32. Gu K*, et al.* Defect-Rich High-Entropy Oxide Nanosheets for Efficient 5-Hydroxymethylfurfural Electrooxidation. *Angew. Chem. Int. Ed.* **60**, 20253-20258 (2021).
