# Supplementary material for: The role of oxygen-vacancy in bifunctional indium oxyhydroxide catalysts for electrochemical coupling of biomass valorization with CO2 conversion
Source: Nat Commun. 2023 Apr 11;14:2040. doi: 10.1038/s41467-023-37679-3 (PMC10090200; doi:10.1038/s41467-023-37679-3)
Supplement: Supplementary file 2 — Description of Additional Supplementary Files [file 41467_2023_37679_MOESM2_ESM.pdf]

Dataset 1: Input file of calculations (INCAR).

Dataset 2: CONTCAR file of the converged calculations for InOOH.

Dataset 3: CONTCAR file of the converged calculations for InOOH-Ov.
